# Radiosynthesis and *In Vitro* Evaluation of [^18^F]FE-MPC6827 as a Candidate PET Tracer for Imaging Microtubule Dynamics

**DOI:** 10.3390/cells15212033

**Published:** 2026-11-07

**Authors:** Michael Gemki, Laura Wunderlich, Laura Köhler, Felix Neumaier, Heike Endepols, Boris D. Zlatopolskiy, Bernd Neumaier, Hans Zempel

**Affiliations:** 1 Institute of Neuroscience and Medicine, Nuclear Chemistry (INM-5), Forschungszentrum Jülich GmbH, Wilhelm-Johnen-Straße, 52428 Jülich, Germany; 2 Institute of Radiochemistry and Experimental Molecular Imaging, Faculty of Medicine and University Hospital Cologne, University of Cologne, Kerpener Straße 62, 50937 Cologne, Germany; 3 Department of Nuclear Medicine, University Hospital RWTH Aachen, Pauwelsstraße 30, 52074 Aachen, Germany; 4 Institute of Human Genetics, Faculty of Medicine and University Hospital Cologne, University of Cologne, Kerpener Straße 34, 50931 Cologne, Germany; 5 Center for Molecular Medicine Cologne (CMMC), University of Cologne, Robert-Koch-Straße 21, 50931 Cologne, Germany; 6 Department of Nuclear Medicine, Faculty of Medicine and University Hospital Cologne, University of Cologne, Kerpener Straße 62, 50937 Cologne, Germany; 7 Center for Human Genetics and Genomic Medicine, Medical Faculty, University Hospital RWTH Aachen, 52062 Aachen, Germany

**Keywords:** microtubule dynamics, radiofluorination, positron emission tomography tracer, *in vitro* studies

## Abstract

**Highlights:**

**What are the main findings?**

- An improved radiosynthesis affords [^18^F]FE-MPC6827 in high radiochemical yields and purities.
- Cellular tracer uptake increases with microtubule destabilization and inversely correlates with markers of microtubule stability.

**What are the implications of the main findings?**

- [^18^F]FE-MPC6827 is a promising radiofluorinated candidate for PET imaging of microtubule dynamics in the central nervous system.
- Further studies are required to confirm its binding specificity and evaluate its *in vivo* imaging properties.

**Abstract:**

Microtubule (MT) dysfunction is a hallmark of neurodegenerative diseases, tauopathies, and genetic disorders. Positron emission tomography (PET) enables the non-invasive assessment of MT dynamics *in vivo*, but current ^11^C-labeled radiotracers are limited by the short half-life of carbon-11. Here, we report the radiosynthesis and *in vitro* evaluation of [^18^F]FE-MPC6827, an ^18^F-labeled derivative of the brain-penetrant MT-destabilizing agent MPC-6827 (verubulin). Radiosynthesis from the corresponding tosylate precursor afforded [^18^F]FE-MPC6827 in 60 ± 3% radiochemical yields (*n* = 22), >99% radiochemical purities, and molar activities up to 475 GBq/µmol. Physicochemical profiling revealed a log*D*_7.4_ value of 3.37 ± 0.05 and favorable CNS-MPO, BBB, and CNS-PET-MPO scores, although brain permeability remains to be established experimentally. In HEK293T cells, MT destabilization significantly increased tracer accumulation, whereas MT stabilization significantly reduced uptake. Overexpression of MT-associated proteins (tau, spastin, MARK, α-tubulin) produced directionally consistent but statistically non-significant changes in tracer uptake, concordant with its effects on MT mass or stability. Across the experiments, tracer uptake correlated inversely with the markers of MT stability. These findings suggest responsiveness to changes in MT organization, although preferential binding to depolymerized tubulin remains to be confirmed by direct binding studies. Taken together, [^18^F]FE-MPC6827 is a promising first-in-class radiofluorinated PET tracer candidate for imaging MT dynamics in the central nervous system.

## 1. Introduction

Microtubules (MT) are cytoskeletal filaments formed by the non-covalent polymerization of α- and β-tubulin heterodimers into protofilaments, which associate laterally to form hollow cylindrical structures [1]. These dynamic structures undergo continuous assembly and disassembly at their plus ends, a process that allows for rapid reorganization of the cytoskeleton and is regulated by GTP hydrolysis, MT-associated proteins (e.g., tau), and MT-severing enzymes (e.g., spastin), as well as post-translational modifications (PTMs) [2,3,4,5,6]. MTs are essential for cell division, maintain the cellular architecture and enable the intracellular transport of organelles, vesicles, or other cargos [7,8]. In neurons, MT integrity is indispensable for axodendritic maintenance and long-range vesicular trafficking, both of which are critical for synaptic plasticity and neuronal homeostasis [6].

Due to their central role in cell division, MTs represent established targets for antimitotic agents such as vincristine, vinblastine, or paclitaxel (Taxol) [9,10,11,12,13], and are increasingly investigated for their utility as diagnostic markers of proliferative activity [14]. In addition, emerging evidence indicates that disruption of MT dynamics could be an early and potentially causative event in the pathogenesis of neurodegenerative diseases such as Alzheimer’s disease (AD) [15] and Parkinson’s disease (PD) [16]. Thus, impaired MT function may contribute to tau hyperphosphorylation in AD, axonal degeneration in PD, and the pathogenic mechanisms of several genetic disorders [17,18]. Disease-associated mutations in genes encoding the key regulators of MT dynamics, including the ATPase-deficient spastin K388R variant, further highlight the central role of cytoskeletal dysregulation in hereditary spastic paraplegia and related neurodevelopmental disorders [19]. Changes in *α*-tubulin expression or PTMs can affect the dynamic equilibrium between polymerized and soluble tubulin pools, altering MT stability and turnover [20]. The microtubule-associated protein tau binds and stabilizes MTs, promoting their longevity and maintaining proper axonal transport. Aberrant phosphorylation of tau by kinases such as microtubule affinity-regulating kinase 2 (MARK2) alters its MT-binding capacity, contributing to pathological MT depolymerization and loss of neuronal polarity, both of which are features observed in multiple models of neurodegenerative diseases [21,22].

**Figure 1:**
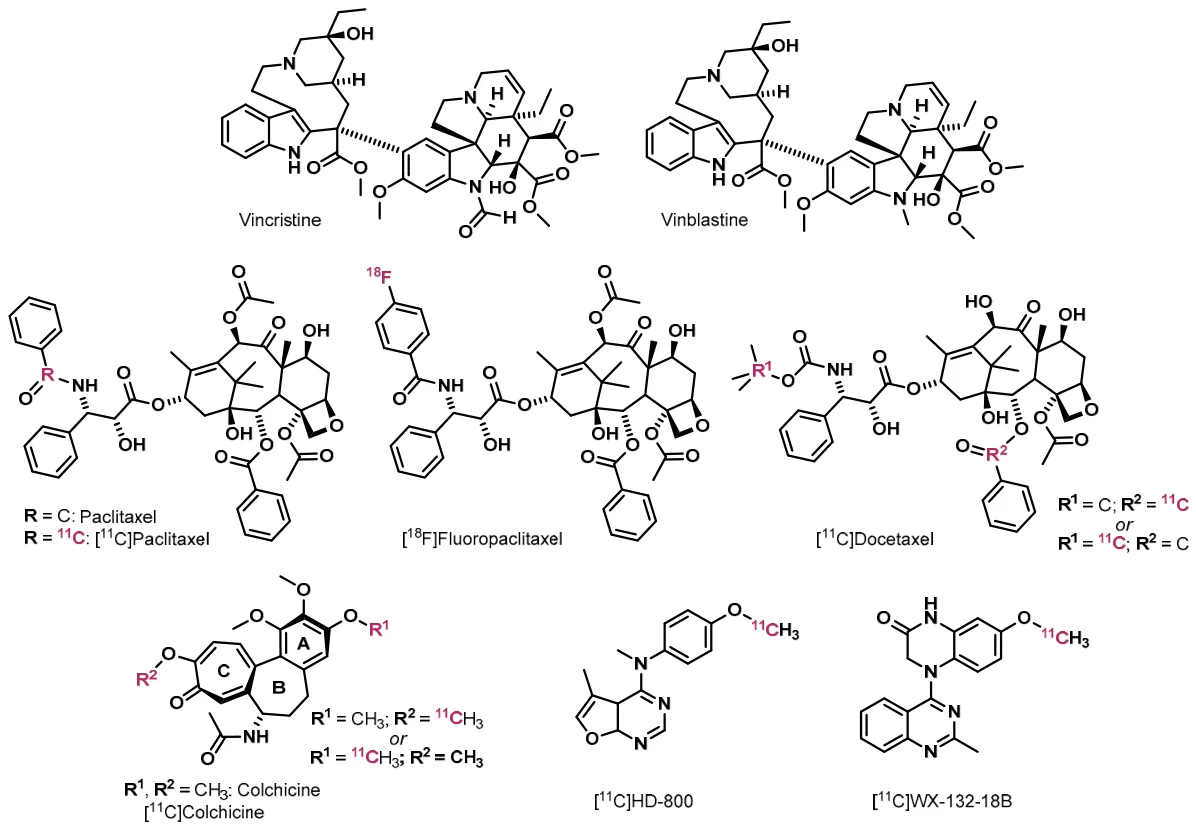
Structure of microtubule-targeting agents and radiotracer candidates for PET imaging of microtubule dynamics. For colchicine, the bonds between rings A and C are drawn in perspective to indicate the axial chirality of natural (−)-colchicine, which possesses the (a*R*,7*S*) configuration.

Given the central role of MTs in numerous physiological and pathological processes, *in vivo* imaging of MT integrity and dynamics has gained attention as a potential strategy for early diagnosis and mechanistic understanding of neurodegenerative disorders [23]. Accordingly, radiolabeled derivatives of established MT-targeting drugs have been explored for positron emission tomography (PET) imaging of MT dynamics [23] (Figure 1).

**Figure 2:**
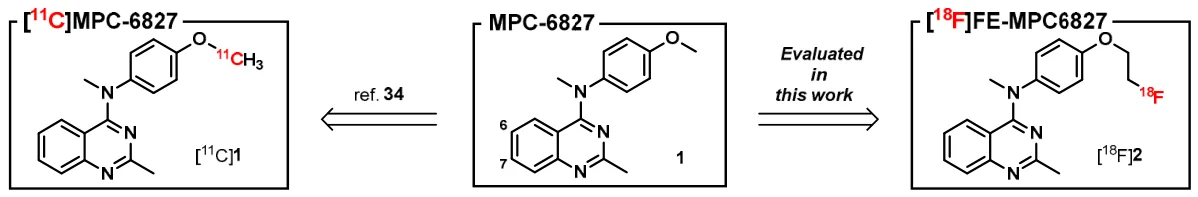
Structure of MPC-6827 (verubulin, **1**), its ^11^C-labeled isotopologue ([^11^C]**1**) and the ^18^F-fluoroethylated derivative [^18^F]**2** evaluated in the present work.

However, first-generation MT PET tracers targeting either the taxane binding site on the *β*-tubulin subunit (e.g., [^11^C]paclitaxel [24], [^18^F]fluoropaclitaxel [25,26], and [^11^C]docetaxel [27]) or the colchicine binding site on the tubulin intra-dimer interface (e.g., [^11^C]colchicine [28,29]) exhibited limited brain uptake due to active efflux via P-glycoprotein (P-gp) and other transporters at the blood-brain barrier (BBB). To overcome this limitation, second-generation compounds with improved central nervous system (CNS) penetration, such as [^11^C]HD-800 [30,31], [^11^C]WX-132-18B [32,33] and [^11^C]MPC-6827 ([^11^C]verubulin) [34,35,36], have been developed. Among these, [^11^C]MPC-6827 (Figure 2) shows particularly promising properties, such as high brain uptake in mice [34] and non-human primates [35,37], excellent metabolic stability [36], high specific binding [34], and very few off-targets in high-throughput screens [34]. Moreover, and in contrast to taxane-based agents, MPC-6827 has been reported to retain its activity in cancer cell lines overexpressing multidrug resistance transporters, including P-gp, suggesting reduced susceptibility to transporter-mediated efflux [38]. Consistent with this favorable profile, [^11^C]MPC-6827 has demonstrated substantial brain uptake in preclinical PET studies [39,40,41]. [^11^C]MPC-6827 binds to the colchicine site at the intra-dimer interface between *α*- and *β*-tubulin subunits of unassembled tubulin heterodimers [42,43], which should result in preferential association with depolymerized MT pools. In line with this assumption, previous studies have demonstrated significantly higher binding of [^11^C]MPC-6827 to the depolymerized tubulin fraction in SH-SY5Y neuroblastoma cells and a significant reduction in brain uptake in healthy mice after treatment with the BBB-penetrant MT-stabilizing agent epothilone D [39]. Additionally, brain uptake and/or the cerebral distribution of [^11^C]MPC-6827 have been shown to be altered in tau knockout mice and murine or non-human primate models of neurodegenerative diseases [39,40,41], suggesting that it can detect neurodegeneration-associated changes in MT dynamics. However, despite the favorable imaging characteristics of [^11^C]MPC-6827, its widespread application is inherently limited by the short physical half-life of carbon-11 (t_1/2_ = 20 min), which necessitates on-site cyclotron access and restricts its use to specialized centers. In contrast, fluorine-18 offers several practical advantages for clinical translation, including excellent accessibility in the form of no-carrier-added (n.c.a.) [^18^F]fluoride ([^18^F]F^−^), a lower maximum positron energy than carbon-11 (E_βmax_ = 0.634 MeV vs. 0.960 MeV), which facilitates high-resolution PET imaging, and a half-life (t_1/2_ = 110 min) compatible with centralized radiotracer production and distribution. As such, the evaluation of ^18^F-labeled analogs of MPC-6827 represents a logical next step towards enabling broader access to MT imaging and enhancing the translational potential of this class of radiotracers.

Here, we report the radiosynthesis and preclinical *in vitro* evaluation of [^18^F]FE-MPC6827 ([^18^F]**2**) (Figure 2), an ^18^F-fluoroethylated derivative of MPC-6827 (**1**) that could combine the promising pharmacokinetic features of [^11^C]MPC-6827 ([^11^C]**1**) with the logistical and imaging advantages of fluorine-18. Although [^18^F]FE-MPC6827 has been briefly mentioned in the patent literature and reported to show brain penetration and a cerebral distribution comparable to [^11^C]MPC-6827 in rodents [44], detailed data on its radiosynthesis, physicochemical properties, and interaction with MTs have not yet been published. Using a combination of MT stabilizers (paclitaxel, epothilone D), MT destabilizers (nocodazole, colchicine, vinblastine), and transient overexpression of MT-associated proteins (α-tubulin, tau, spastin, MARK2), we therefore investigated tracer uptake by HEK293T cells under varying cytoskeletal conditions. In addition, homologous blocking studies were performed to evaluate the binding specificity of the radiotracer.

## 2. Materials and Methods

### 2.1. General

Unless noted otherwise, all chemicals and solvents were purchased from VWR International GmbH (Darmstadt, Germany), Sigma-Aldrich Chemie GmbH (Steinheim, Germany), ABCR GmbH (Karlsruhe, Germany), Apollo Scientific Ltd. (Bredbury, UK) or BLD Pharmatech GmbH (Kaiserslautern, Germany) and used without further purification.

Unless stated otherwise, all reactions were carried out with magnetic stirring. Organic extracts were dried over anhydrous MgSO_4_. Air- and/or moisture-sensitive reagents were handled in flame-dried glassware under argon (>99.999%, Air Liquide GmbH, Düsseldorf, Germany). Solutions were concentrated under reduced pressure (1–900 mbar) at 40–50 °C using a rotary evaporator.

#### 2.1.1. Nuclear Magnetic Resonance (NMR) Spectroscopy

NMR spectra (^1^H: 400 MHz, ^13^C{^1^H}: 101 MHz; ^19^F: 376 MHz) were measured at ambient temperature in deuterated solvents as indicated using a Bruker Ascend 400 (Bruker Biospin GmbH, Rheinstetten, Germany). The measured chemical shifts are reported in δ [ppm] relative to residual peaks of non-deuterated solvents. Higher order NMR spectra were approximately interpreted as first-order spectra if possible. The observed signal multiplicities are characterized as follows: s = singlet, bs = broad singlet, d = doublet, m = multiplet, dd = doublet of doublets, and ddd = doublet of doublets of doublets. Coupling constants *J* are reported in hertz (Hz). The ^13^C NMR spectra were acquired using an attached proton test (APT) experiment, in which quaternary carbon and methylene signals exhibit the opposite phase to methine and methyl signals.

#### 2.1.2. Mass Spectrometry

High-resolution electrospray ionization mass spectrometry (HR-ESI-MS) was performed with an LTQ XL™ Orbitrap™ mass spectrometer (Thermo Fisher Scientific Inc., Bremen, Germany). High-resolution gas chromatography electron ionization mass spectrometry (HR-GC-EI/MS) was performed with an Exactive™ GC Orbitrap™ mass spectrometer (Thermo Fisher Scientific Inc., Bremen, Germany).

#### 2.1.3. Column Chromatography

Manual column chromatography was performed with silica gel, 60 Å, 230–400 mesh particle size from VWR International GmbH (Darmstadt, Germany) or silica gel (w/0.1% Ca), 60 Å, 230–400 mesh particle size from Sigma-Aldrich GmbH (Steinheim, Germany). Automated column chromatography was performed an a Büchi Pure C-815 flash system (Büchi Labortechnik GmbH, Essen, Germany) using Reveleris C-18 reversed phase cartridges (Büchi Labortechnik GmbH, Essen, Germany).

#### 2.1.4. Thin-Layer Chromatography

Thin-layer chromatography (TLC) was performed using aluminum sheets coated with silica gel 0.25 mm SIL G/UV 254 (Merck KGaA, Darmstadt, Germany). Chromatograms were inspected under UV light (*λ* = 254 nm) and/or stained with phosphomolybdic acid (4% in EtOH), ninhydrin (0.5% in *n*-butanol), or potassium permanganate solution (0.75% KMnO_4_, 5% K_2_CO_3_, and 0.07% NaOH in H_2_O). Staining was employed as a complementary visualization method for reaction monitoring, particularly for components that could not be reliably detected under UV light.

### 2.2. Syntheses

#### 2.2.1. Synthesis of 4-[Methyl(2-methylquinazolin-4-yl)amino]phenol (Desmethyl-MPC-6827, **4**) [34]

DIPEA (4.96 mL, 3.68 g, 28.5 mmol, 1.25 eq.) was added dropwise to a solution of 4-chloro-2-methylquinazoline (**3**, 4.50 g, 25.2 mmol, 1.10 eq.) and 4-(methylamino)phenol hemisulfate (Metol, 3.90 g, 11.4 mmol [corresponding to 22.8 mmol of 4-(methylamino)phenol], 1.00 eq) in *i*-PrOH (150 mL, 0.15 m with respect to 4-(methylamino)phenol), and the mixture was stirred for 18 h at 80 °C. After cooling to ambient temperature, the precipitate was isolated by filtration, washed with ice-cold *i*-PrOH (3 × 4 mL), and dried under reduced pressure at 80 °C to afford the analytically pure title compound as a fluffy, shiny yellow powder (5.53 g, 20.8 mmol, 92% yield). R*_f_* = 0.23 (CH_2_Cl_2_/MeOH = 15:1). ^1^H NMR [(CD_3_)_2_SO, 400 MHz]: *δ* = 9.88 (bs, 1H), 7.73–7.66 (m, 2H), 7.20–7.14 (m, 3H), 6.88–6.85 (m, 3H), 3.58 (s, 3H), 2.64 (s, 3H). ^13^C NMR [(CD_3_)_2_SO, 101 MHz]: *δ* = 160.79, 160.56, 157.01, 145.87, 137.50, 133.48, 127.34, 126.32, 125.18, 123.18, 116.85, 112.99, 43.21, 24.18. HR-GC-EI/MS: *m*/*z* = [M-H]^•+^ calcd for (C_16_H_14_N_3_O): 264.1131; detected: 264.1131.

#### 2.2.2. Synthesis of *N*-[4-(2-Fluoroethoxy)phenyl]-*N*,2-dimethylquinazolin-4-amine (FE-MPC6827, **2**) [44]

Cs_2_CO_3_ (309 mg, 0.95 mmol, 1.68 eq.) was added to a suspension of **4** (150 mg, 0.57 mmol, 1.00 eq.) in MeCN (3.50 mL, 0.17 m), and the resulting mixture was stirred for 10 min at ambient temperature followed by 1 h at 70 °C. After cooling to 50 °C, 1-fluoro-2-iodoethane (168 µL, 359 mg, 2.06 mmol, 3.65 eq.) was added and the mixture was stirred for another 22 h at 50 °C. The reaction mixture was then cooled to ambient temperature, filtered, and the solvent was removed under reduced pressure. The crude product was purified by column chromatography (CH_2_Cl_2_/MeOH = 25:1) to afford the title compound as a pale-yellow powder (111 mg, 0.36 mmol, 63% yield). R*_f_* = 0.26 (CH_2_Cl_2_/MeOH = 25:1). ^1^H NMR [(CD_3_)_2_SO, 400 MHz]: *δ* = 7.66–7.56 (m, 2H), 7.20–7.17 (m, 2H), 7.08–6.95 (m, 4H), 4.82–4.80 (m, 1H), 4.70–4.68 (m, 1H), 4.30–4.28 (m, 1H), 4.23–4.21 (m, 1H), 3.49 (s, 3H), 2.58 (s, 3H). ^13^C NMR [(CD_3_)_2_SO, 101 MHz]: *δ* = 162.34, 161.02, 156.46, 151.71, 141.33, 131.84, 127.50, 127.24, 125.65, 124.05, 115.85, 114.13, 82.11 (d, *J* = 166.7 Hz), 67.28 (d, *J* = 18.9 Hz), 42.31, 26.12. ^19^F NMR [(CD_3_)_2_SO, 376 MHz]: *δ* = -222.10 (s). HR-GC-EI/MS: *m*/*z* = [M-H]^•+^ calcd for (C_18_H_17_FN_3_O): 310.1350; detected: 310.1349.

#### 2.2.3. Synthesis of 2-{4-[Methyl(2-methylquinazolin-4-yl)amino]phenoxy}ethyl 4-Methylbenzenesulfonate (**5**) [44]

Cs_2_CO_3_ (1.47 g, 4.53 mmol, 4.0 eq.) was added to a suspension of **4** (300 mg, 1.13 mmol,1.00 eq.) in MeCN (22.0 mL, 0.05 m), and the resulting mixture was stirred for 10 min at ambient temperature followed by 90 min at 70 °C. After slight cooling, 1,2-bis-tosyloxyethane (837 mg, 2.26 mmol, 2.00 eq.) was added and the mixture was stirred for another 22 h at 70 °C. The reaction mixture was then cooled to ambient temperature, filtered, and the solvent was removed under reduced pressure. The crude product was purified by column chromatography (CH_2_Cl_2_/MeOH = 25:1) to afford the title compound as a sticky, highly hygroscopic yellow powder (283 mg, 0.61 mmol, 54% yield). R*_f_* = 0.25 (CH_2_Cl_2_/MeOH = 25:1). ^1^H NMR [(CD_3_)_2_SO, 400 MHz]: *δ* = 7.83–7.81 (d, *J* = 8.2 Hz, 2H), 7.66–7.63 (dd, *J* = 8.5, 1.4 Hz, 1H), 7.60–7.56 (ddd, *J* = 8.4, 6.7, 1.5 Hz, 1H), 7.49–7.47 (d, *J* = 8.2 Hz, 2H), 7.17–7.15 (d, *J* = 8.9 Hz, 2H), 7.07–7.03 (ddd, *J* = 8.4, 6.7, 1.5 Hz, 1H), 6.95–6.92 (dd, *J* = 8.5, 1.4 Hz, 1H), 6.90–6.88 (d, *J* = 8.9 Hz, 2H), 4.36–4.34 (m, 2H), 4.19–4.17 (m, 2H), 3.48 (s, 3H), 2.58 (s, 3H), 2.41 (s, 3H). ^13^C NMR [(CD_3_)_2_SO, 101 MHz]: *δ* = 162.32, 161.00, 156.03, 151.62, 145.02, 141.39, 132.25, 131.87, 130.16, 127.68, 127.44, 127.16, 125.63, 124.07, 115.84, 114.10, 69.10, 65.57, 42.30, 26.10, 21.08. HR-ESI-MS: *m*/*z* = [M+H]^+^ calcd for (C_25_H_25_N_3_O_4_S): 464.1638; detected: 464.1637.

### 2.3. Radiochemistry

#### 2.3.1. General Conditions

[^18^F]Fluoride ([^18^F]F^−^) was produced by bombardment of enriched [^18^O]H_2_O with 17 MeV protons in a BC1710 cyclotron (The Japan Steel Works, Tokyo, Japan) or a GE PETtrace (GE Healthcare, Chicago, IL, USA), both at the INM-5 (Forschungszentrum Jülich GmbH, Jülich, Germany). Radioactivity was measured using a CRC-55tR Dose Calibrator (Capintec Inc., Florham Park, The Netherlands) and/or a Curiementor 2 (PTW, Freiburg, Germany). The following cartridges were used for radiosyntheses and solid phase extractions (SPEs): F-18 separation cartridges Sep-Pak™ Plus Light QMA Carbonate cartridges (130 mg or 46 mg sorbent, preconditioned with 1 mL H_2_O) from Waters GmbH (Eschborn, Germany) and Oasis^®^ Plus Light HLB extraction cartridges (30 mg sorbent, preconditioned with 3 mL EtOH and 10 mL H_2_O) from Waters GmbH (Eschborn, Germany).

The identity of radiolabeled products was confirmed by co-injection of the corresponding non-radioactive reference compounds. Radiochemical yields (RCYs) corrected for decay to the start of synthesis and/or activity yields (AY; non-decay-corrected) are provided for analytically pure radiolabeled compounds. Radiochemical conversions (RCCs) were determined by HPLC analysis after dilution of the reaction mixtures with HPLC mobile phase. To this end, the decay corrected product peak areas in the chromatograms were compared to the decay corrected peak area corresponding to a post-column injection (p.c.i.) [45]. All radiosyntheses were carried out according to the radiation protection rules of our institute using appropriate personal protective equipment and shielding. Unless noted otherwise, 5 mL screw top V-Vials (20–400 thread, 20 × 65 mm) were used for all radiosyntheses. Prior to their first use and after every 10–15 reactions, the vials were (re)siliconized to minimize adsorption of [^18^F]F^−^ to the vessel walls. To this end, the vials were incubated for 10 min with a solution of 5% SiCl_2_Me_2_ in CH_2_Cl_2_. Following incubation, the vials were sequentially rinsed with EtOAc, CH_2_Cl_2_, acetone, H_2_O, and MeOH. During the H_2_O and acetone rinsing steps, mechanical assistance with a lint-free paper towel was applied to enhance cleaning efficiency. Finally, the vials were dried at 90 °C for 10 min prior to (re)use.

#### 2.3.2. Manual Preprocessing of [^18^F]F^−^

Aqueous [^18^F]F^−^ was loaded (from the male side) onto a QMA cartridge using a 1 mL syringe, the cartridge was washed (from the male side) with MeOH (2 mL) to remove residual H_2_O, and dried (from the female side) for 2 min in a stream of argon. The [^18^F]F^−^ was then eluted from the female side with an appropriate phase transfer catalyst in MeOH, as described in Section 2.3.4 (*Note*: If the QMA cartridge is loaded, flushed, and eluted from the female side only, a significant amount of [^18^F]F^−^ remains on the resin).

#### 2.3.3. Analytical High-Performance Liquid Chromatography (HPLC)

HPLC analyses were carried out on a Vanquish^®^ Analytical Purification LC System, equipped with a VC-D11-A-01 Diode Array Detector CG and a VC-P20-A-01 Quaternary Pump C (Thermo Fisher Scientific Inc., Bremen, Germany), coupled in series with a Berthold LB500 NaI detector, giving a time delay of 0.1–0.2 min (depending on flow-rate) between the corresponding responses. Two Rheodyne 6 port injection valves equipped with equal sample loops were installed before and behind the column. Columns and methods are given for each chromatogram separately.

#### 2.3.4. Manual Radiosynthesis of [^18^F]FE-MPC6827 ([^18^F]**2**)

Following the preprocessing described in Section 2.3.2, [^18^F]F^−^ was eluted from the QMA cartridge with a solution of Bu_4_NOTs (1 mg, 2.42 µmol) in MeOH (1 mL) and collected in a 5 mL screw-top reaction V-vial equipped with a septum cap. After evaporation of the solvent and addition of precursor **5** (0.5 mg, 1.1 µmol) in MeCN (800 µL) through the septum using a 1 mL syringe, the reaction mixture was stirred under argon for 10 min at 110 °C using a magnetic stir bar. After cooling to ambient temperature, the reaction was quenched by addition of HPLC mobile phase [4 mL, 30% MeCN (0.1% TFA)] and the product was isolated by semipreparative reversed phase HPLC with a Waters XBridge BEH Shield RP18 (5 µm, 130 Å, 250 × 10 mm) column at a flow rate of 4.7 mL/min. The purified product-fraction was diluted with H_2_O (100 mL) and loaded onto a HLB cartridge, which was washed with H_2_O (15 mL) and dried in a stream of argon for 2 min. The product was eluted from the cartridge with EtOH (1.5 mL) followed by evaporation of the solvent under dynamic vacuum (250 mbar) in a stream of argon at 85 °C for 5 min. The residue was formulated in 100 µL of a vehicle consisting of aqueous Captisol^®^ stock solution (25%, *w*/*v*, 70 µL), EtOH (10 µL), and polyethylene glycol (PEG-400; 20 µL), corresponding to a Captisol^®^ concentration of 17.5% (*w*/*v*), 10% (*v*/*v*) EtOH, and 20% (*v*/*v*) PEG-400 before further dilution with physiological saline as required. Using this protocol, [^18^F]FE-MPC6827 ([^18^F]**2**) was obtained in radiochemical conversions of 95 ± 3% (*n* = 28), radiochemical yields of 60 ± 3% (*n* = 22), and activity yields of 43 ± 6%, within an overall synthesis time of 90 ± 5 min. The radiochemical purity at the end of synthesis (EOS) exceeded 99% and molar activities ranged from 253 to 475 GBq/µmol for starting activities between 3 and 6 GBq.

#### 2.3.5. Determination of Lipophilicity

The lipophilicity of [^18^F]FE-MPC6827 was determined using a shake-flask partitioning method. Briefly, aliquots of the radiotracer in EtOH (1–2 µL) were added to mixtures of *n*-octanol and phosphate-buffered saline (PBS, pH = 7.4) or water (750 µL of each) in 2 mL Eppendorf vials. The vials were vortexed for 2 min and centrifuged for another 2 min to separate the two phases. Aliquots of the upper *n*-octanol phase (400 µL) were then carefully removed from the top, while aliquots of the aqueous phase (400 µL) were removed by piercing the bottom of the vial with a syringe and cannula to avoid contact with the organic phase. Both fractions were transferred into fresh vials and counted in a gamma counter to determine the activity in the organic and aqueous phases. Partition and distribution coefficients were calculated as the logarithm of the ratio of the decay-corrected radioactivity concentrations in the organic and aqueous phases. Measurements were performed in two independent experiments with a total of eight determinations.

### 2.4. In Vitro Evaluation

#### 2.4.1. Cell Culture and Reagents

Human embryonic kidney 293T (HEK293T) cells were cultured under standard growth conditions (37 °C in a humidified atmosphere containing 5% CO_2_ and 95% air) in Dulbecco’s Modified Eagle Medium supplemented with GlutaMAX™ (DMEM; Gibco, Waltham, MA, USA), 10% fetal bovine serum (Gibco), and 1% antibiotic-antimycotic solution. Cells were routinely passaged twice a week.

#### 2.4.2. Transfection

HEK293T cells were seeded at a density of 2 × 10^5^ cells per well in 12-well plates (1 mL medium per well) for uptake assays and at a density of 5 × 10^4^ cells per well in 24-well plates (0.5 mL medium per well) for immunocytochemistry on coverslips coated with poly-d-lysine (PDL) (0.5 mL medium per well). Transfection complexes were prepared by mixing 500–1000 ng of plasmid DNA with polyethyleneimine (PEI; 3 µL per 1 µg DNA) and diluted in Opti-MEM medium (Gibco) to a final volume of 200 µL for uptake assays or 50 µL for immunostaining experiments. The DNA-PEI mixture was incubated at room temperature for 20 min and then added dropwise to the cells. The cultures were kept at 37 °C in a humidified atmosphere with 5% CO_2_ for 24 h prior to downstream analysis.

#### 2.4.3. Pharmacological Treatment

To modulate MT dynamics pharmacologically, HEK293T cells were seeded on PDL-coated 24-well plates with coverslips at a density of 5 × 10^4^ cells per well. Cells were incubated for 6 h with the MT-stabilizing agents paclitaxel (Thermo Fisher Scientific Inc.) or epothilone D (Cayman Chemical Company, Ann Arbor, MI, USA), or with the MT-destabilizing agents nocodazole (Merck Millipore, Darmstadt, Germany), colchicine (Cytogen GmbH, Greven, Germany), or vinblastine (Sigma Aldrich GmbH), each at a final concentration of 1 µM, which were added in serum- and antibiotic-free DMEM supplemented with GlutaMAX™. DMSO at 0.1% served as vehicle control.

#### 2.4.4. Immunofluorescence Staining

MT stability was assessed by immunofluorescence detection of tyrosinated and acetylated tubulin. Transfected and treated HEK293T cells grown on PDL-coated coverslips were fixed with 3.7% formaldehyde in PBS and washed with PBS. The cells were then permeabilized and blocked for 10 min at room temperature in PBS containing 5% bovine serum albumin (Sigma Aldrich GmbH) and 0.2% Triton X-100 (AppliChem GmbH, Darmstadt, Germany). After another wash, the coverslips were incubated with the respective primary antibody (Anti-alpha-Tubulin Antibody, tyrosinated, clone YL1/2, 1:1000, #MAB1864-I, LOT: 4270985, Merck Millipore; Acetyl-α-Tubulin (Lys40) (D20G3) XP^®^ Rabbit mAb, 1:1000, #5335, LOT: 5, Cell Signaling) for 3 h at ambient temperature, washed, and then incubated with the corresponding *AlexaFluor* coupled secondary antibody (Thermo Fisher Scientific Inc.) for 2 h under the same conditions. The cell nuclei were stained with NucBlue™ Live Cell Reagent (Invitrogen, Carlsbad, CA, USA). Finally, the coverslips were mounted on glass slides and allowed to dry at ambient temperature for 24 h prior to imaging. The immunofluorescence signals were obtained using the Axio Imager.Z2m microscope equipped with Axiocam 705 Mono (Zeiss, Oberkochen, Germany).

#### 2.4.5. Image Analysis

For quantitative analysis with Fiji/ImageJ (version 1.54p, Java 1.8.0_322, 64-bit; National Institutes of Health, Bethesda, MD, USA), a somatic region of interest (ROI) was manually defined per cell, excluding the cell nucleus. A background ROI was positioned next to each cell, and background fluorescence was subtracted from the raw cell signal. Only transfected cells identified by GFP fluorescence encoded by the plasmid constructs were evaluated. The mean fluorescence intensities of acetylated and tyrosinated tubulin were quantified and normalized to controls (DMSO-treated cells for pharmacological treatments; cells transfected with GFP only for transfections). For statistical evaluation, ten cells per condition were analyzed in three independent biological experiments (*n* = 3). Fluorescence intensity data are presented as mean ± standard error of the mean (SEM). To assess potential treatment-associated cell death, DAPI-stained fluorescence microscopy images of HEK293T cells subjected to pharmacological treatment were additionally analyzed based on nuclear morphology. Nuclei exhibiting morphological features indicative of cell death were counted, and their proportion relative to the total number of evaluated nuclei was determined. Three replicates were analyzed per treatment condition (*n* = 3). Further details are provided in Section S6 of the Supporting Information. Statistical analyses were performed using GraphPad Prism 10. Differences between groups were evaluated by one-way ANOVA followed by Dunnett’s post hoc multiple-comparisons test against the respective control group. Statistical significance was defined as follows: ns (*p* > 0.05), * (*p* ≤ 0.05), ** (*p* ≤ 0.01), *** (*p* ≤ 0.001), and **** (*p* ≤ 0.0001).

#### 2.4.6. Cellular Uptake Studies

For cellular uptake assays, 2 × 10^5^ HEK293T cells were seeded in PDL-coated 12-well plates and cultured in 1 mL of serum- and antibiotic-free DMEM supplemented with GlutaMAX™. Cells were incubated with 112.5 kBq [^18^F]FE-MPC6827 for 20, 40, 60, or 80 min at 37 °C in a humidified 5% CO_2_ atmosphere, and uptake was quantified as described below. Based on the results, an incubation time of 1 h was selected for subsequent treatment and transfection experiments. To pharmacologically modulate MT dynamics, cells were treated with either MT-stabilizing agents (1 µm paclitaxel or 1 µm epothilone D) or MT-destabilizing agents (1 µm vinblastine, 1 µm nocodazole, or 1 µm colchicine). Drug treatments were performed for 6 h prior to tracer incubation. Following treatment, [^18^F]FE-MPC6827 was added and cells were incubated for 1 h at 37 °C. In parallel, tracer uptake was evaluated in HEK293T cells transiently transfected with GFP-tagged constructs encoding α-tubulin, tau, spastin (wild-type or ATPase-deficient K388R mutant), or MARK2 to genetically alter MT stability. To assess tracer specificity, a subset of cells was pretreated for 1 h with 50 µm non-radioactive FE-MPC6827 before incubation with the radiotracer under identical conditions. Following incubation, the supernatant was collected, and the cells were washed twice with PBS before being lysed in 40 mm NaOH. Radioactivity in both the supernatant and cell fractions was quantified using a gamma counter. Measured values were normalized to the seeded cell number and subsequently normalized to the mean uptake and corresponding control condition (DMSO for pharmacological treatments or GFP for transfection experiments). For transfection experiments, uptake values were corrected for an average transfection efficiency of approximately 25%, which was determined by quantifying the ratio of GFP-positive to total cells in representative microscopy images. The correction was applied by adjusting the difference between each construct and the GFP control by a factor of four to account for the fraction of transfected cells (non-normalized data presented in Supplemental Figure S4). All data are presented as mean ± SEM from three independent biological experiments, each measured in triplicate wells. Statistical analyses were conducted using GraphPad Prism 10. Differences between groups were evaluated by one-way ANOVA followed by Dunnett’s post hoc multiple-comparisons test against the respective control group. Statistical significance was defined as follows: ns (*p* > 0.05), * (*p* ≤ 0.05), ** (*p* ≤ 0.01), *** (*p* ≤ 0.001), and **** (*p* ≤ 0.0001). Correlation analyses were performed using the Pearson product-moment correlation coefficient in Minitab 17.1.0 (Minitab Inc., State College, PA, USA).

## 3. Results and Discussion

### 3.1. Chemistry

**Scheme 1:**
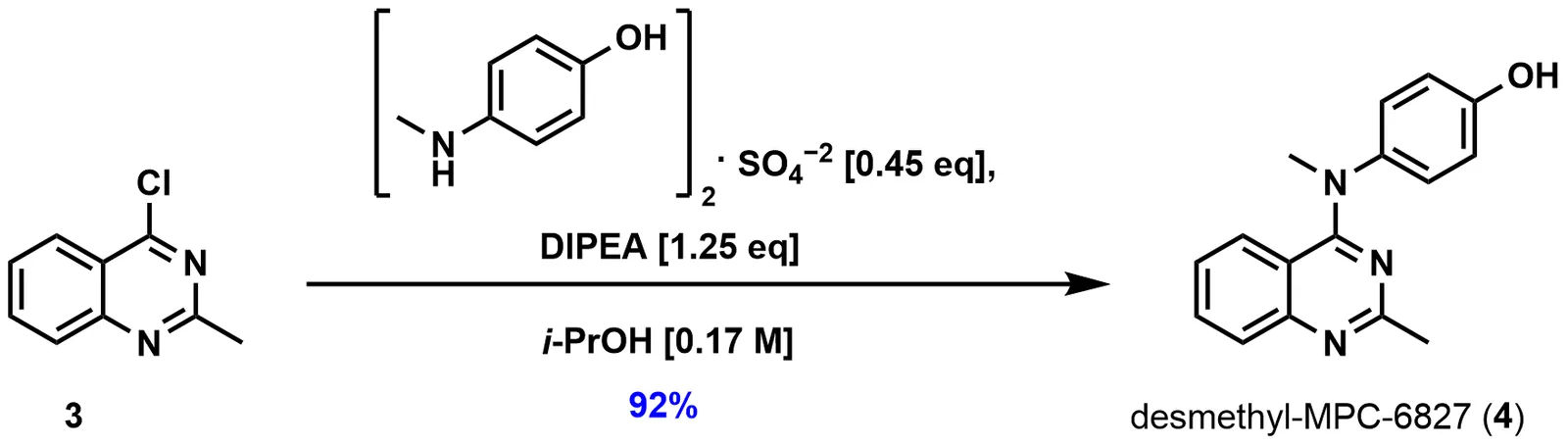
Synthesis of the key-intermediate desmethyl-MPC-6827 (**4**). Reagents and conditions: 4-(Methylamino)phenol hemisulfate (Metol), DIPEA, *i*-PrOH, 80 °C, 18 h. Abbreviations: DIPEA = *N*,*N*-diisopropylethylamine.

**Scheme 2:**
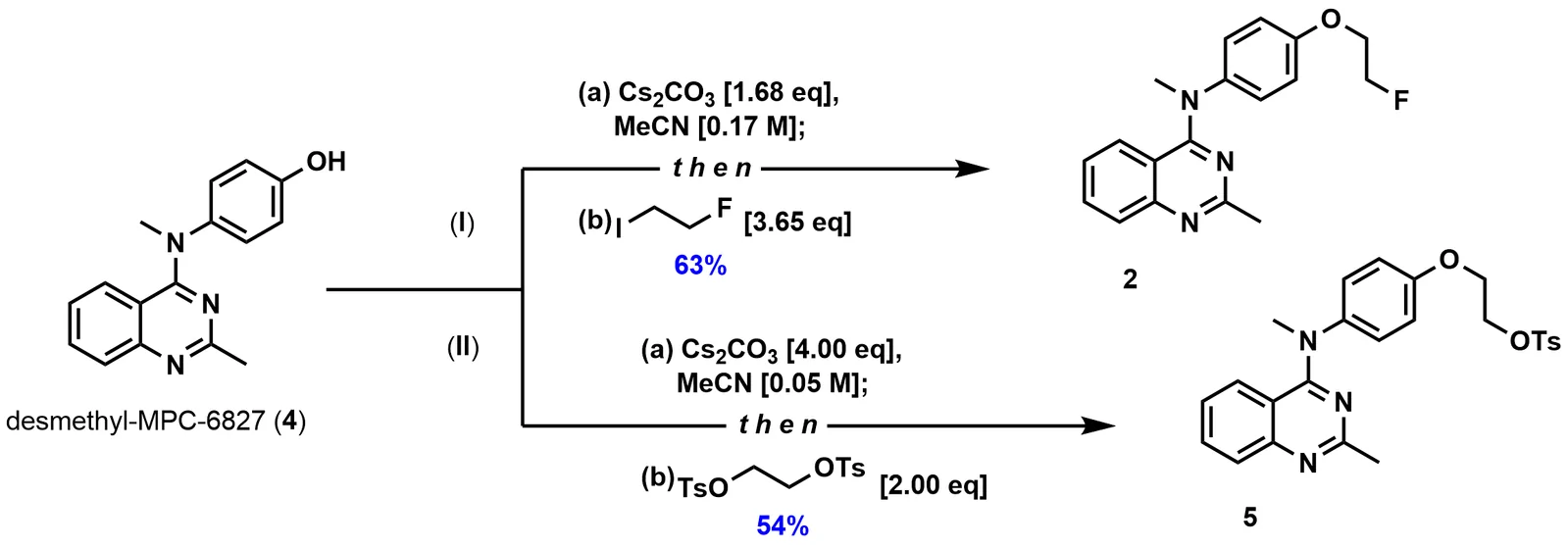
Synthesis of non-radioactive reference compound **2** and radiolabeling precursor **5**. Reagents and conditions: (I-a) Cs_2_CO_3_, MeCN, 70 °C, 60 min; then: (I-b) 70 °C → 50 °C, 1-fluoro-2-iodoethane, 50 °C, 22 h; (II-a) Cs_2_CO_3_, MeCN, 70 °C, 90 min; then: (II-b) 70 °C → 50 °C, 1,2-bis(tosyloxy)ethane, 70 °C, 22 h.

Since the synthesis of the central intermediate desmethyl-MPC-6827 (**4**) via *O*-demethylation of commercially available MPC-6827 with BBr_3_ has been shown to result in contamination with trace amounts of MPC-6827 [34], we adopted an alternative synthetic approach designed to improve chemical purity. Specifically, the desired fluorinated reference compound **2,** as well as the radiolabeling precursor **5** (Scheme 1 and Scheme 2), were prepared by a minor modification of the protocol reported by Willardsen and co-workers [46].

#### 3.1.1. Synthesis of the Reference Compound

As outlined in Scheme 1, intermediate **4** was prepared by coupling commercially available 4-chloro-2-methylquinazoline (**3**) with 4-(methylamino)phenol hemisulfate (Metol) in the presence of *N*,*N*-diisopropylethylamine (DIPEA) for 18 h at 80 °C. The reaction proceeds through nucleophilic aromatic substitution (S*_N_*Ar) at the electron-deficient C4 position of the quinazoline ring and involves displacement of the chlorine substituent in **3** by the methylamino group of Metol to form the desired 4-aminoquinazoline derivative **4**. The reaction mixture was then cooled to ambient temperature, and the resulting precipitate was isolated by filtration, washed with ice-cold *i*-PrOH, and dried under reduced pressure at 80 °C to afford **4** in excellent yield (92%) and sufficient purity for use without further purification. To access the non-radioactive reference compound **2** (Scheme 2-I), **4** was deprotonated with Cs_2_CO_3_ at 70 °C, followed by *O*-alkylation with 1-fluoro-2-iodoethane for 22 h at 50 °C, which delivered the desired reference compound in 63% yield.

#### 3.1.2. Precursor Synthesis

The radiolabeling precursor **5** was synthesized by *O*-alkylation of **4** with 1,2-bis(tosyloxy)ethane under conditions similar to those described above (Scheme 2-II). Subsequent purification by reversed-phase column chromatography afforded the desired tosylate precursor **5** in a satisfactory yield of 54%.

#### 3.1.3. Radiosynthesis

**Scheme 3:**
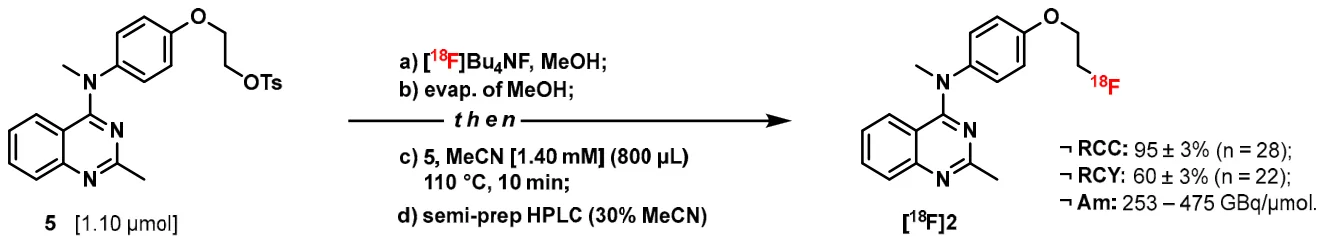
Radiosynthesis of [^18^F]FE-MPC6827 ([^18^F]**2**). Reagents and conditions: (a) elution of [^18^F]F^−^ with Bu_4_NOTs (2.42 µmol) in MeOH (1 mL); (b) evaporation of MeOH; (c) addition of precursor **5** (1.10 µmol) in MeCN (800 µL), argon, 110 °C, 10 min; (d) *column*: XBridge BEH Shield RP18, 5 µm (130 Å), 250 × 10 mm (Waters, Vienna, Austria); *eluent*: 30% MeCN (0.1% TFA); *flow-rate*: 4.7 mL/min. Abbreviations: Bu_4_NOTs = tetrabutylammonium tosylate, TFA = trifluoroacetic acid.

[^18^F]FE-MPC6827 ([^18^F]**2**) was prepared by aliphatic radiofluorination of precursor **5** using Bu_4_NOTs for [^18^F]F^−^ elution according to the protocol developed by Krasikova *et al.* [47] (Scheme 3). The radiofluorination reaction proceeds through nucleophilic aliphatic substitution (S*_N_*2), in which [^18^F]F^−^ displaces the terminal tosylate leaving group of precursor **5**. To this end, aqueous [^18^F]F^−^ was trapped on a QMA cartridge and eluted with Bu_4_NOTs in MeOH. After evaporation of the solvent and addition of precursor **5** (1.10 µmol) in MeCN (800 µL), the reaction mixture was stirred for 10 min at 110 °C under argon (Scheme 3). Thereafter, [^18^F]**2** was isolated by HPLC and formulated with aqueous Captisol^®^ (25%, *w*/*v*) in EtOH/polyethylene glycol (PEG-400) (1:2) to obtain an injectable solution. The sulfobutylether-β-cyclodextrin derivative Captisol^®^ was selected as a solubilizing excipient to facilitate formulation of the relatively lipophilic radiotracer in a vehicle suitable for experimental administration. The initial formulation (100 µL) consisted of 70% aqueous Captisol^®^ stock solution (25%, *w*/*v*), 10% (*v*/*v*) EtOH, and 20% (*v*/*v*) PEG-400, corresponding to a final Captisol^®^ concentration of 17.5% (*w*/*v*) before further dilution with physiological saline as required. The EtOH concentration of 10% (*v*/*v*) refers to the initial formulation before further dilution with physiological saline as required, which reduced the EtOH concentration accordingly. Although this formulation was suitable for the present experimental studies, its suitability for clinical administration, including the tolerability of the excipients and stability upon dilution, remains to be established. Further formulation optimization may therefore be necessary before clinical translation.

Using this protocol, the tracer was obtained in radiochemical conversions of 95 ± 3% (*n* = 28), radiochemical yields of 60 ± 3% (*n* = 22), and activity yields of 43 ± 6%, within an overall synthesis time of 90 ± 5 min. The radiochemical purity at the end of synthesis (EOS) exceeded 99% and molar activities ranged from 253 to 475 GBq/µmol (for starting activities between 3 and 6 GBq). These molar activities would correspond to approximately 10.5–19.8 pmol (3.30–6.20 ng) of FE-MPC6827 for a hypothetical administered activity of 5 MBq, suggesting that relatively low carrier masses can be achieved for preclinical PET applications. Nevertheless, their suitability in terms of target occupancy and potential pharmacological effects remains to be established.

Although the radiosyntheses reported in the present study were performed manually, preliminary experiments have demonstrated the feasibility of transferring the procedure to an automated synthesis module. However, further optimization and validation will be necessary to establish a standardized automated production process for future preclinical and potential clinical applications.

### 3.2. Lipophilicity and MPO Scores

Lipophilicity is a key determinant of a tracer’s pharmacokinetic properties, influencing passive diffusion across the BBB, systemic clearance, and non-specific binding. It is typically characterized by the partition coefficient (log*P*) or the distribution coefficient at physiological pH (log*D*_7.4_), measured between an aqueous phase and an organic solvent such as 1-octanol. To assess these properties for [^18^F]FE-MPC6827, experimental log*P* and log*D*_7.4_ values were determined by the “shake-flask method” [48] and compared to calculated values generated using the ADMETlab 3.0 platform [49] (Table 1). The experimental and predicted log*D*_7.4_ value were in close agreement and fell within the range generally considered optimal for efficient BBB penetration and favorable target-to-background ratios (2.0–3.5). In contrast, the experimental log*P* value was approximately two log units lower than the predicted value, and, unexpectedly, also lower than the measured log*D*_7.4_. This discrepancy may partly arise from differences in the experimental conditions, as log*P* was determined using unbuffered water, whereas log*D*_7.4_ was measured in PBS (pH 7.4). For potentially ionizable compounds, measurements in unbuffered water may not accurately reflect the intrinsic partition coefficient of the neutral species. In addition, differences in aqueous-phase composition and methodological effects associated with radioactivity-based shake-flask measurements may influence the results [48]. Although the values were reproducible across two independent experiments (*n* = 8), systematic effects cannot be excluded, and the origin of the discrepancy remains unresolved. Further experimental verification under controlled pH conditions is therefore required before definitive conclusions regarding the intrinsic lipophilicity of [^18^F]FE-MPC6827 can be drawn.

To further contextualize the physicochemical properties of the tracer candidate in terms of its suitability for brain imaging, we performed multiparameter optimization (MPO) assessments. Consistent with previous reports of effective BBB penetration [44], [^18^F]FE-MPC6827 achieved a CNS-MPO score [50] of 5.0, a BBB score [51] of 4.8 and a CNS-PET-MPO score [52] of 3.8 (see Table S1, Supporting Information), all of which exceed commonly used thresholds for sufficient brain permeability and/or low non-specific binding.

Although the calculated CNS-MPO, BBB, and CNS-PET-MPO scores suggest physicochemical properties compatible with brain penetration, these predictions require experimental validation. Furthermore, the predictive performance of these *in silico* approaches has not been specifically validated for the present fluoroethylated 4-aminoquinazoline scaffold, and their results do not account for all factors influencing CNS PET tracer performance, including active efflux, metabolic stability, and non-specific tissue binding. Initial investigations could include permeability measurements using a parallel artificial membrane permeability assay for the BBB (PAMPA-BBB), complemented by cellular transport studies to assess potential interactions with efflux transporters such as P-gp [53]. Ultimately, *in vivo* PET imaging and radiometabolite analyses will be necessary to characterize brain uptake, retention, and metabolic stability.

### 3.3. In Vitro Evaluation: Relationship between Microtubule Stability and [^18^F]FE-MPC6827 Uptake

#### 3.3.1. Immunofluorescence Analysis of Microtubule Stability

To validate the suitability of the HEK293T cell model for assessing MT dynamics, we first examined how pharmacological and genetic manipulations influence MT stability in this model. These experiments aimed to establish a controlled system in which changes in MT mass and turnover could be quantified, supporting the interpretation of subsequent tracer uptake studies. HEK293T cells cultured on poly-d-lysine (PDL)-coated coverslips were immunostained for acetylated α-tubulin as a marker for stabilized MTs, and tyrosinated α-tubulin as a marker for dynamic MTs.

Treatment with the MT-stabilizing agents paclitaxel and epothilone D both increased the intensity of acetylated α-tubulin staining (2.834 ± 0.701 and 8.862 ± 1.081, respectively), although only the effect of epothilone D reached statistical significance. Since acetylation of α-tubulin occurs predominantly in long-lived MTs and serves as an indicator of reduced MT turnover [54,55], these results indicate robust MT stabilization by epothilone D, whereas paclitaxel may be a less effective stabilizer in HEK293T cells. A modest increase in tyrosinated α-tubulin was also observed (1.125 ± 0.211 for paclitaxel and 1.293 ± 0.192 for epothilone D), suggesting partial remodeling of dynamic MTs.

**Figure 3:**
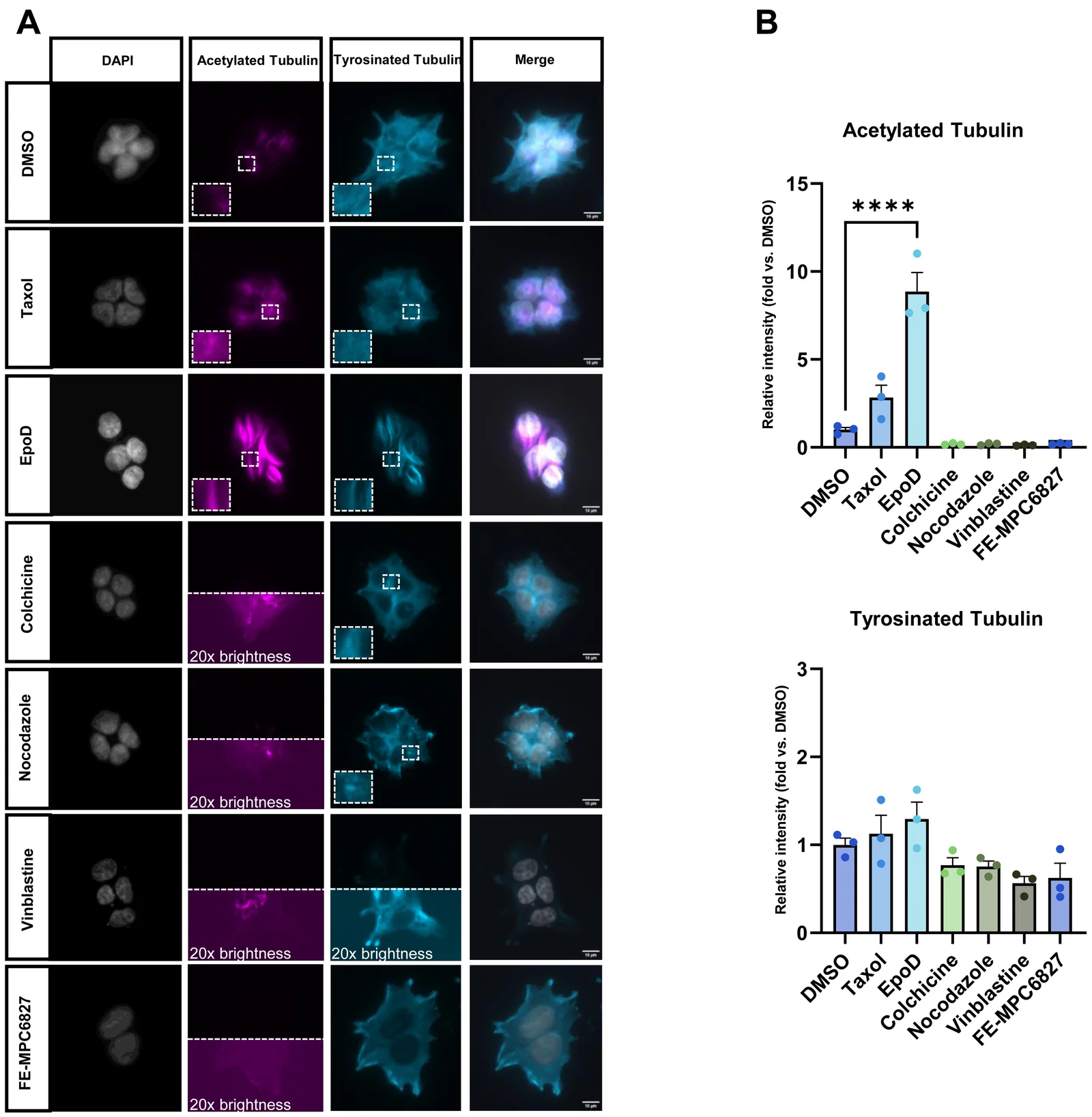
Effects of pharmacological microtubule stabilization or destabilization on markers of microtubule dynamics in HEK293T cells. (**A**) Representative immunofluorescence images of HEK293T cells treated with DMSO, paclitaxel (Taxol), epothilone D (EpoD), colchicine, nocodazole, or vinblastine. Cells were treated for 6 h before they were fixed with 3.7% paraformaldehyde, and immunostained for acetylated α-tubulin, tyrosinated α-tubulin, and nuclei (DAPI). Merged images illustrate the overall microtubule organization under each condition. Scale bar: 10 µm. (**B**) Quantification of fluorescence intensities for acetylated and tyrosinated α-tubulin. One-way ANOVA revealed a significant overall treatment effect for acetylated α-tubulin (F(6,14) = 43.08, *p* < 0.0001). Dunnett’s post hoc test showed that epothilone D significantly increased acetylated tubulin levels relative to DMSO (adjusted *p* < 0.0001), whereas paclitaxel, colchicine, nocodazole, vinblastine, and FE-MPC6827 showed no significant effects. For tyrosinated α-tubulin, one-way ANOVA also detected a significant overall effect (F(6,14) = 3.889, *p* = 0.0170), but no individual treatment differed significantly from DMSO in Dunnett’s multiple-comparison test (all adjusted *p* > 0.16). Data are presented as mean ± SEM (*n* = 10 cells per condition; 3 independent experiments). Fluorescence intensities were normalized to DMSO controls. White dashed boxes indicate regions shown as enlarged insets, whereas horizontal dashed lines delineate image areas displayed at 20-fold increased brightness to improve the visualization of weak fluorescence signals. Statistical significance was determined using one-way ANOVA with comparison of each treatment to DMSO controls. Significance: *p* ≤ 0.0001 (****).

In contrast, exposure to the MT-destabilizing agents colchicine, nocodazole, and vinblastine resulted in a marked decrease in acetylated α-tubulin (0.182 ± 0.032, 0.186 ± 0.032, and 0.122 ± 0.016, respectively). Tyrosinated α-tubulin levels also declined modestly (0.770 ± 0.085, 0.754 ± 0.062, and 0.564 ± 0.076), indicating that these compounds induce broad MT depolymerization affecting both stable and dynamic subpopulations in HEK293T cells (Figure 3B).

These findings support the interpretation that MT-destabilizing agents promote net cytoskeletal breakdown rather than a compensatory increase in dynamic MTs.

Consistent with the MT-destabilizing properties of its parent compound MPC-6827 [38], treatment with 1 µm non-radioactive FE-MPC6827 also reduced acetylated α-tubulin staining (0.198 ± 0.027) and moderately decreased tyrosinated α-tubulin levels (0.624 ± 0.167), further confirming the sensitivity of HEK293T cells to compound-induced alterations in MT integrity.

To investigate whether treatment-associated cell death might have influenced the observed changes in tracer accumulation, we additionally performed a morphology-based assessment of nuclear changes in pharmacologically treated HEK293T cells (Figure S6, Supporting Information).

The proportion of cells exhibiting nuclear morphological features indicative of cell death ranged from 6% to 13% across the investigated conditions, compared with 7% in the DMSO-treated controls. Only nocodazole treatment resulted in a statistically significant increase relative to the control. These findings suggest that treatment-associated cell death alone is unlikely to account for the observed differences in tracer uptake, although potential effects of cell detachment or changes in the number of viable adherent cells cannot be excluded.

To investigate whether genetic modulation of MT stability produces effects comparable to those of the pharmacological interventions, HEK293T cells were transfected with GFP-tagged constructs encoding selected MT-associated proteins. These included α-tubulin, tau, wild-type spastin, an ATPase-deficient spastin mutant (K388R), and the kinase MARK2, which are known to influence MT polymerization, severing, or stabilization through distinct mechanisms.

**Figure 4:**
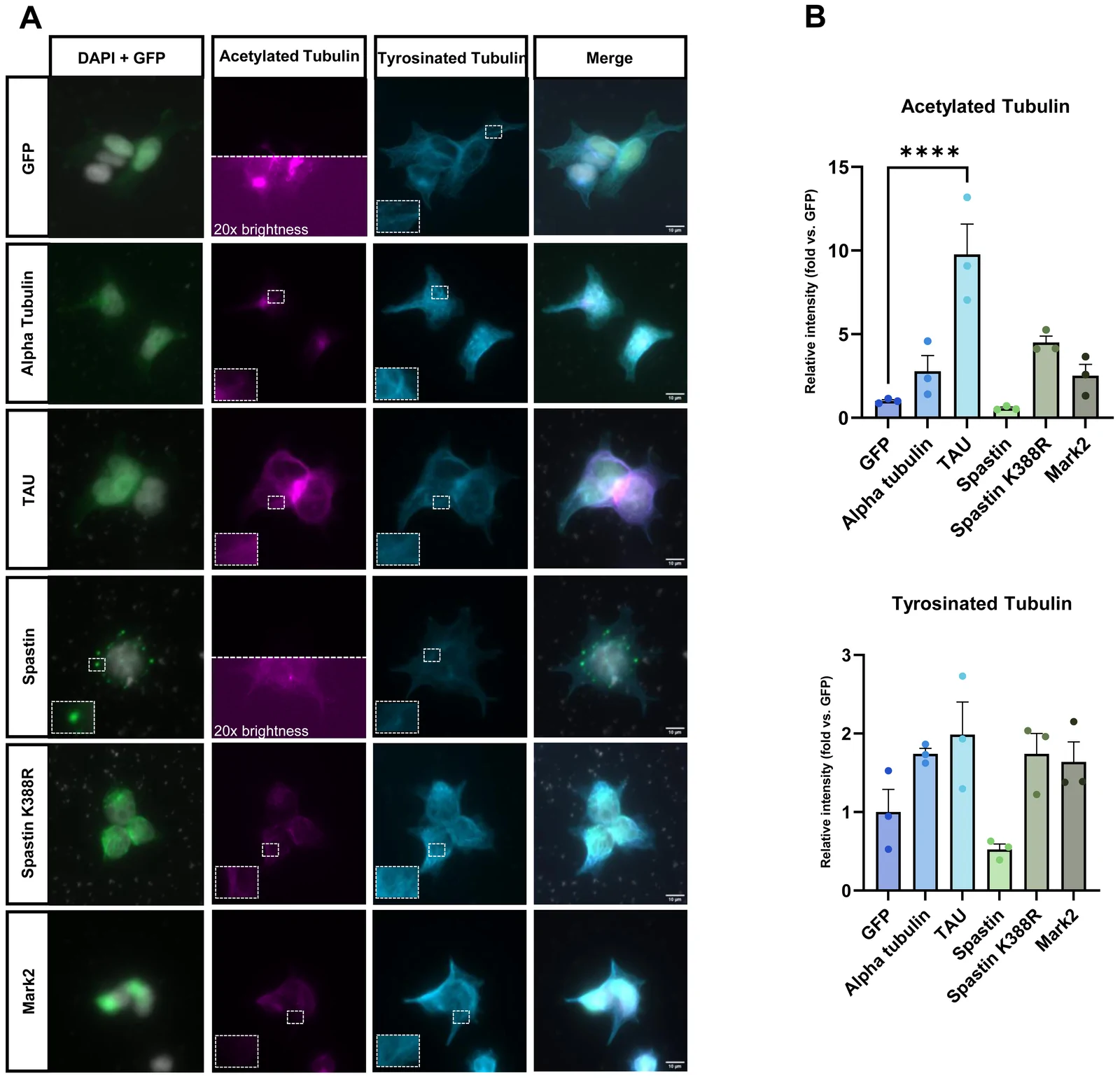
Effects of microtubule-associated protein overexpression on markers of microtubule dynamics in HEK293T cells. (**A**) Representative immunofluorescence images of HEK293T cells transfected with GFP, α-tubulin, tau, wild-type spastin, ATPase-deficient spastin (K388R), and MARK2 for 24 h, fixed with 3.7% paraformaldehyde, and immunostained for acetylated α-tubulin, tyrosinated α-tubulin, and nuclei (DAPI). GFP fluorescence was used to identify transfected cells. Merged images illustrate characteristic changes in microtubule organization upon overexpression of individual constructs. Scale bar: 10 µm. (**B**) Quantification of acetylated and tyrosinated α-tubulin intensities. One-way ANOVA revealed a significant overall effect of transfection on acetylated tubulin levels (F(5,12) = 14.27, *p* = 0.0001). Dunnett’s post hoc test indicated a significant increase only for tau (adjusted *p* < 0.0001), whereas α-tubulin, spastin, spastin K388R, and MARK2 showed no significant differences relative to GFP. For tyrosinated tubulin, one-way ANOVA also showed a significant main effect (F(5,12) = 4.666, *p* = 0.0135); however, no construct differed significantly from GFP in Dunnett’s post hoc comparisons (all adjusted *p* > 0.05). Data represent mean ± SEM (*n* = 10 cells per condition; 3 independent experiments). Fluorescence intensities were normalized to GFP controls. White dashed boxes indicate regions shown as enlarged insets, whereas horizontal dashed lines delineate image areas displayed at 20-fold increased brightness to improve the visualization of weak fluorescence signals. Statistical significance was determined using one-way ANOVA with comparison of each treatment to GFP controls. Significance: *p* ≤ 0.0001 (****).

The immunofluorescence analysis revealed that overexpression of α-tubulin, tau, and the ATPase-deficient spastin mutant (K388R) increased both acetylated and tyrosinated α-tubulin staining, with a stronger effect on acetylated α-tubulin, suggesting a shift toward overall MT stabilization with residual dynamic activity [56,57] (Figure 4). Among these, tau overexpression significantly increased acetylated α-tubulin (9.770 ± 1.807) and modestly elevated tyrosinated α-tubulin (1.986 ± 0.415) in a non-significant manner, consistent with its role in promoting MT stabilization. α-Tubulin (2.779 ± 0.941 and 1.741 ± 0.069) and spastin (K388R) (4.505 ± 0.375 and 1.741 ± 0.260) produced directionally consistent but non-significant increases in acetylated and tyrosinated α-tubulin, respectively. While α-tubulin overexpression appeared to slightly shift the balance toward polymerization, expression of the ATPase-deficient spastin mutant (K388R) produced stronger effects on acetylated α-tubulin levels, supporting the idea that loss of spastin cleavage activity promotes excessive MT stabilization [19].

In contrast, the overexpression of wild-type spastin, a MT-severing AAA-ATPase, reduced both acetylated (0.574 ± 0.069) and tyrosinated (0.525 ± 0.070) α-tubulin levels. Although these effects did not reach statistical significance, they are consistent with spastin’s established role in promoting MT turnover [58].

Finally, the expression of MARK2, a kinase known to phosphorylate tau and other MT-associated proteins, resulted in a moderate, non-significant increase in both acetylated (2.519 ± 0.678) and tyrosinated (1.639 ± 0.256) α-tubulin staining, indicating a mild stabilizing effect (Figure 4B). Although MARK2 is classically known for destabilizing MTs through phosphorylation of tau [59], this finding may reflect cell type-specific differences in MARK2 signaling or compensatory effects resulting from transient overexpression in HEK293T cells. MARK2 has been shown to be involved in various signaling pathways that regulate cytoskeletal organization and could indirectly contribute to MT stabilization under certain conditions [22]. Therefore, the trend toward increased α-tubulin acetylation observed in our model may represent indirect or secondary effects of MARK2 activity rather than a direct phosphorylation-dependent destabilization of tau-bound MTs.

Overall, genetic modulation induced more subtle and variable effects on MT stability than pharmacological manipulations, likely reflecting differences in the magnitude and localization of cytoskeletal remodeling achievable through transient protein overexpression in HEK293T cells.

#### 3.3.2. Cellular Uptake Studies: Pharmacological Modulation of Microtubule Stability Alters Tracer Accumulation

**Figure 5:**
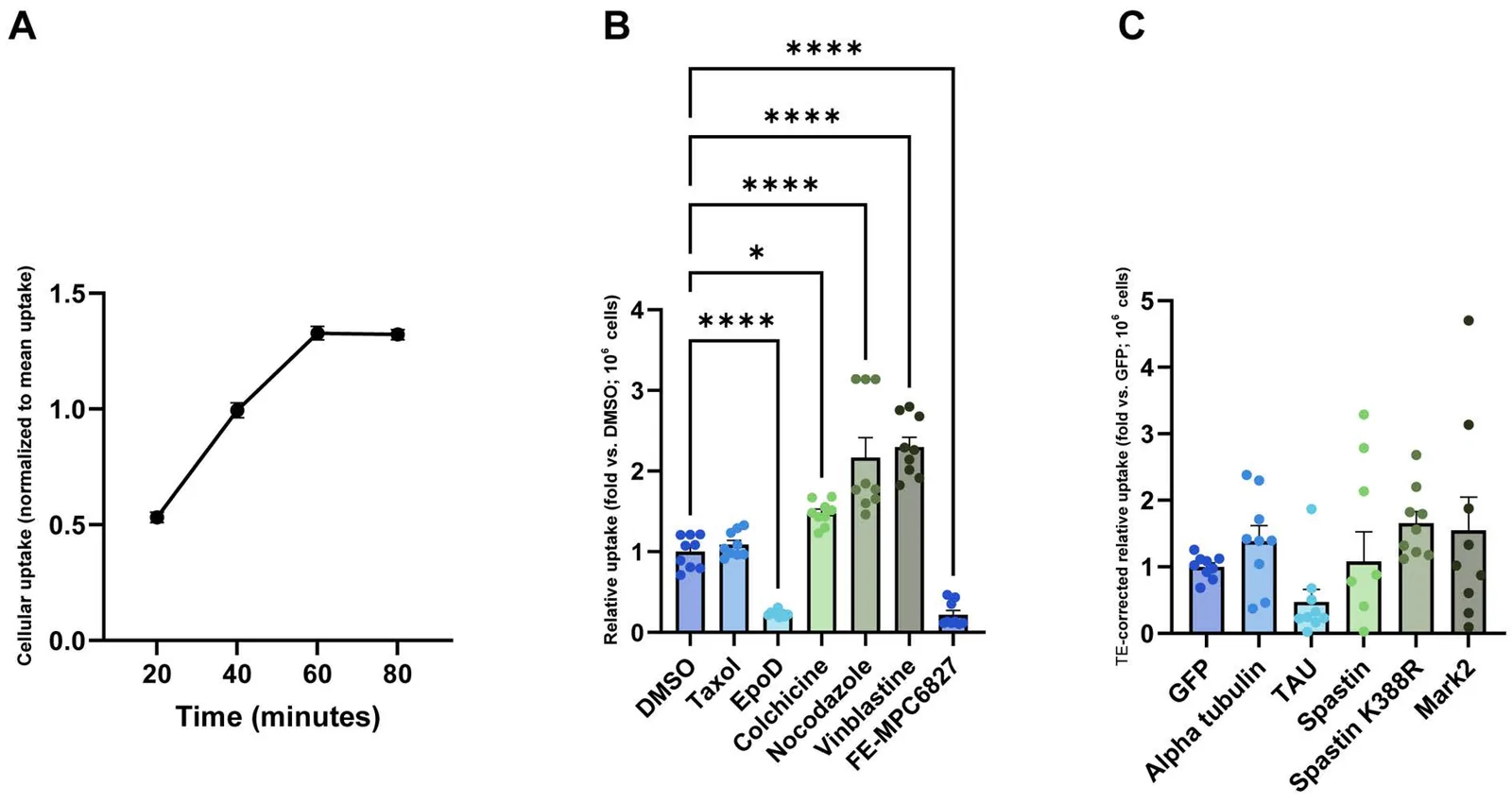
Modulation of [^18^F]FE-MPC6827 uptake by pharmacologic or genetic manipulation of microtubule dynamics. (**A**) Time-dependent uptake of [^18^F]FE-MPC6827 ([^18^F]**2**) measured after 20, 40, 60, and 80 min. Tracer accumulation increased over time and reached a plateau at 60 min, which was used as standard incubation duration for all further experiments. (**B**) Effect of pharmacological modulation and homologous blocking on tracer uptake. Treatment with the microtubule-stabilizing agent epothilone D (EpoD) significantly reduced tracer accumulation, whereas paclitaxel (Taxol) showed no effect compared to DMSO controls. In contrast, the microtubule-destabilizing agents colchicine, nocodazole, and vinblastine all significantly increased tracer uptake. Pretreatment with non-radioactive FE-MPC6827 (50 µm) caused a significant decrease in uptake. (**C**) Effect of genetic modulation of microtubule stability on tracer accumulation. Overexpression of α-tubulin, tau, wild-type spastin, ATPase-deficient spastin (K388R), or MARK2 did not significantly alter uptake relative to GFP controls, although a minor downward trend was observed for tau. Based on microscopic evaluation, an average transfection efficiency (TE) of approximately 25% was achieved. Uptake values were therefore displayed on a fourfold adjusted scale to account for the lower proportion of transfected cells within each well. Data represent mean ± SEM (3 technical replicates; 3 independent biological experiments). Values were normalized to the mean cell uptake, control conditions (DMSO or GFP), and seeded cell number. Statistical significance was determined using one-way ANOVA with comparison of each treatment to DMSO or GFP controls. Significance: *p* < 0.05 (*) and *p* ≤ 0.0001 (****).

To optimize the incubation parameters for subsequent cellular uptake studies, we first assessed the time-dependent accumulation of [^18^F]FE-MPC6827 in untreated HEK293T cells over a period of 80 min. Tracer accumulation increased progressively over time until it reached a plateau at 60 min, which was selected as the standard incubation duration for all further experiments (Figure 5A). Next, the impact of pharmacological modulation of MT dynamics was assessed by pretreating cells with different MT-stabilizing or -destabilizing agents. The tracer uptake after pretreatment was expressed relative to the tracer uptake in the DMSO-treated control cells, which revealed a measurable impact of most agents on tracer accumulation (Figure 5B).

Among the MT-stabilizing agents, epothilone D significantly reduced tracer uptake compared to the DMSO-treated controls (0.231 ± 0.012), consistent with a decrease in the pool of dynamic tubulin subunits available for binding. In contrast, paclitaxel treatment resulted in no measurable change relative to DMSO-treated controls (1.090 ± 0.052). Considering that paclitaxel also failed to produce a statistically significant increase in acetylated α-tubulin staining (Figure 3), this finding could reflect insufficient MT stabilization at the applied concentration (1 µm) and exposure time. Thus, the different responses observed following paclitaxel and epothilone D treatment are most readily explained by differences in their effectiveness in stabilizing the MT network under the investigated conditions.

Conversely, the MT-destabilizing agents colchicine (1.478 ± 0.051), nocodazole (2.169 ± 0.245), and vinblastine (2.297 ± 0.123) all significantly increased radiotracer uptake relative to the DMSO-treated controls, consistent with an association between MT destabilization and increased tracer uptake. However, these effects cannot be attributed exclusively to an increased availability of unpolymerized tubulin heterodimers, as the pharmacological agents differ in their binding sites and mechanisms of action. In particular, colchicine binds to the colchicine-binding site at the α/β-tubulin interface, which is also targeted by the parent compound MPC-6827 [60]. Thus, colchicine treatment may simultaneously increase the pool of depolymerized tubulin and reduce the accessibility of potential binding sites through competitive occupancy. Nocodazole also interacts with the colchicine-binding region and may similarly influence tracer binding [60,61], whereas vinblastine acts at a distinct binding site [60]. Accordingly, differences in tracer accumulation among these agents may reflect a combination of altered MT organization, target availability, and binding-site occupancy.

In addition, homologous blocking studies were performed using a large excess (50 µm) of non-radioactive FE-MPC6827, which significantly reduced tracer uptake (0.219 ± 0.051) compared to the control cells (Figure 5B). This concentration was selected to provide a substantial molar excess over the radiotracer in an initial proof-of-concept experiment. However, as only a single blocking concentration was investigated and non-radioactive FE-MPC6827 itself affects MT organization, the observed reduction should be interpreted as evidence of a substantial homologous-compound-sensitive component of tracer accumulation rather than definitive proof of target-specific binding.

Finally, to assess whether genetic modulation of MT stability exerts effects comparable to the pharmacological manipulations, tracer uptake was analyzed in HEK293T cells transfected with GFP-tagged constructs encoding α-tubulin (1.386 ± 0.234), tau (0.474 ± 0.186), wild-type spastin (1.081 ± 0.447), ATPase-deficient spastin (K388R) (1.656 ± 0.176), or MARK2 (1.549 ± 0.500) (Figure 5C). Although no statistically significant differences were observed between transfected cells and GFP controls, a numerical reduction in tracer uptake was apparent in tau-overexpressing cells, consistent with the direction expected from its MT-stabilizing function. In contrast, overexpression of α-tubulin, spastin (wild-type or K388R), or MARK2 produced much less marked effects.

To further explore the relationship between MT dynamics and tracer accumulation, we analyzed the correlation between changes in [^18^F]FE-MPC6827 uptake and alterations in acetylated or tyrosinated α-tubulin staining observed under the different experimental conditions. Specifically, relative fluorescence intensities of acetylated or tyrosinated α-tubulin (normalized to respective controls) were compared to normalized tracer uptake across all pharmacological and/or genetic manipulations (excluding homologous blocking with non-radioactive FE-MPC6827).

**Figure 6:**
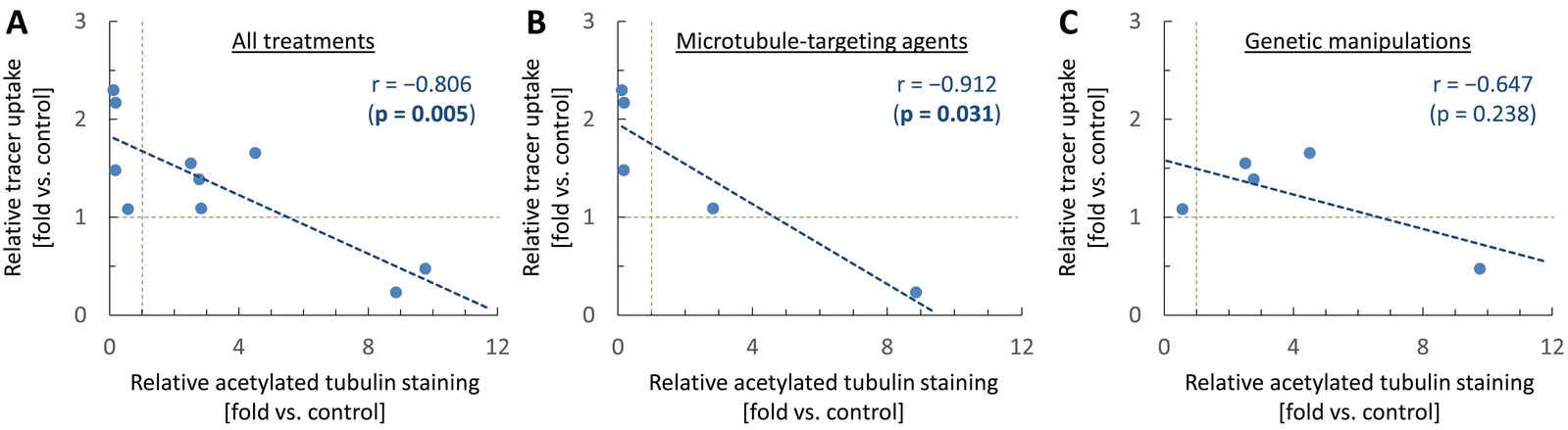
Correlation between changes in [^18^F]FE-MPC6827 uptake and changes in acetylated α-tubulin levels observed in HEK293T cells under different experimental conditions. (**A**) Scatter plot showing the inverse correlation between normalized tracer uptake and acetylated α-tubulin fluorescence intensity across all pharmacological and genetic manipulations (excluding homologous blocking with non-radioactive FE-MPC6827). (**B**) Subgroup analysis showing the correlation for the pharmacological treatments only. (**C**) Subgroup analysis showing the correlation for the genetic manipulations only. Each point represents the mean of three biological replicates. Dark blue dashed lines represent the linear regression fits, whereas light gray dashed lines indicate the reference values of 1 (fold vs. control) on the respective axes. Pearson correlation coefficients (r) and *p*-values are indicated in each panel.

When all the conditions were considered, tracer uptake showed a statistically significant inverse correlation with acetylated α-tubulin levels (Figure 6A), consistent with an association between tracer accumulation and MT stability. A similar but much weaker and non-significant trend was observed for tyrosinated *α*-tubulin (Figure S5A, Supporting Information).

When analyzed separately, the inverse correlations were most pronounced within the pharmacological treatment group for both acetylated (Figure 6B) and tyrosinated (Figure S5B, Supporting Information) α-tubulin, consistent with an association between tracer accumulation and pharmacologically induced changes in MT stability. In contrast, no significant correlation was observed for the genetic manipulations alone (Figure 6C and Figure S5C, Supporting Information).

Taken together, these findings further underscore the sensitivity of [^18^F]FE-MPC6827 to shifts in MT stability and support its potential as a PET radiotracer for imaging dynamic cytoskeletal remodeling in the CNS. The development of [^18^F]FE-MPC6827 builds on previous investigations of colchicine-site-targeting PET tracers, including [^11^C]HD-800 [30,31], [^11^C]WX-132-18B [32,33], and [^11^C]MPC-6827 [34,35,36,37]. These ^11^C-labeled compounds have demonstrated brain penetration in preclinical PET studies, supporting the general feasibility of targeting MT-associated binding sites in the central nervous system. In particular, [^11^C]MPC-6827 has shown promising cerebral uptake and sensitivity to pharmacological modulation of MT stability in animal models. The principal potential advantage of [^18^F]FE-MPC6827 lies in the longer physical half-life and lower positron energy of fluorine-18, which may facilitate broader availability and improved imaging logistics. Furthermore, the radiosynthesis described here provides the tracer in high radiochemical yields and molar activities. Nevertheless, whether the fluoroethylated derivative retains the favorable biological properties of MPC-6827 remains to be established, as structural modification may affect its binding characteristics and pharmacokinetic behavior.

#### 3.3.3. Limitations

Several limitations should be considered when interpreting the present findings. First, although the pharmacological modulation of MT stability significantly altered [^18^F]FE-MPC6827 accumulation, the molecular binding specificity of the fluorinated derivative has not yet been independently established. In particular, competitive interactions with MT-destabilizing agents and potential treatment-related changes in cell viability may influence cellular tracer uptake. Although an additional morphology-based assessment revealed only relatively small differences in the proportion of cells exhibiting nuclear features indicative of cell death across treatment conditions (Figure S6, Supporting Information), this analysis does not account for potential cell detachment. Moreover, differences in the number of viable adherent cells cannot be excluded, particularly because tracer uptake was normalized to the initially seeded cell number. Second, the biological evaluation was restricted to HEK293T cells, and the limited sample size, relatively low transfection efficiency, and assumptions underlying the transfection-efficiency correction constrain interpretation of the genetic-modulation experiments. Furthermore, the discrepancy between the apparent octanol/water lipophilicity value determined using unbuffered water and the measured log*D*_7.4_ requires further investigation and independent verification under controlled pH conditions. Likewise, although preliminary post-synthesis stability assessments suggested that [^18^F]FE-MPC6827 remains stable in its final formulation for up to 24 h, systematic stability studies under defined storage conditions have not yet been performed. Such investigations will be necessary to establish an appropriate shelf life and support future production scale-up and potential clinical translation. Finally, although the computational physicochemical assessments support further development, the BBB permeability, metabolic stability, susceptibility to defluorination or active efflux, and cerebral binding specificity of [^18^F]FE-MPC6827 remain to be established experimentally. Addressing these limitations through complementary binding studies, validation in neuronal models, and *in vivo* PET investigations will be essential for assessing the tracer’s potential for imaging microtubule dynamics in the central nervous system.

## 4. Conclusions

In this study, we developed a robust and efficient protocol for the production of [^18^F]FE-MPC6827, an ^18^F-labeled derivative of the BBB-penetrant MT-destabilizing agent MPC-6827, in yields and molar activities suitable for preclinical evaluation. The physicochemical profiling revealed a favorable log*D*_7.4_ value and promising CNS-MPO, BBB, and CNS-PET-MPO scores, supporting further investigation of [^18^F]FE-MPC6827 as a candidate for CNS PET imaging applications.

In HEK293T cells, the uptake of [^18^F]FE-MPC6827 showed the expected responses to pharmacological modulation of MT stability. MT destabilization with nocodazole, vinblastine, or colchicine significantly increased tracer accumulation, whereas MT stabilization with epothilone D significantly reduced uptake. Genetic manipulation through transient overexpression of MT-associated proteins produced no statistically significant effects on tracer accumulation, although a trend towards reduced uptake was observed in tau-overexpressing cells. In addition, there was a significant inverse correlation between the effects on acetylated α-tubulin staining and tracer uptake across all the experimental conditions, particularly for pharmacological manipulations. Collectively, these findings support an association between [^18^F]FE-MPC6827 accumulation and changes in MT stability, consistent with the proposed preference for binding to depolymerized tubulin, although the underlying binding mechanism remains to be confirmed.

Overall, our findings support the further development of [^18^F]FE-MPC6827 as a candidate PET tracer for investigating MT dynamics. If the binding specificity and suitability for brain imaging can be confirmed *in vivo*, this radiotracer could potentially provide a non-invasive approach for investigating MT dysfunction in neurodegenerative and other CNS disorders. In the longer term, MT-targeting PET imaging might contribute to the characterization of disease-associated cytoskeletal alterations, the monitoring of disease progression, or the assessment of therapeutic interventions targeting MT stability. However, several important requirements must be addressed before clinical translation, including confirmation of target-specific binding, assessment of cerebral pharmacokinetics and metabolic stability, evaluation of injected mass effects and potential pharmacological activity, and development of an appropriate formulation and standardized production procedure. Future investigations should therefore focus on comprehensive *in vivo* characterization in suitable preclinical models to establish the feasibility and potential diagnostic value of [^18^F]FE-MPC6827.

## Figures and Tables

**Table 1:** Measured and calculated log*P* and log*D*_7.4_ values.

Value	[^18^F]FE-MPC6827	
	Measured *	ADMETlab
log*P*	1.36 ± 0.02	3.42
log*D*_7.4_	3.37 ± 0.05	3.33

* *n* = 8; two independent experiments.

## Data Availability

The raw data supporting the conclusions of this article will be made available by the authors on request.

## Supplementary Materials

The following supporting information can be downloaded at: https://www.mdpi.com/article/10.3390/cells15212033/s1, Table S1: CNS-MPO, BBB, and CNS-PET-MPO scores for FE-MPC6827 (**2**); Scheme S1: Radiosynthesis of [^18^F]FE-MPC6827 ([^18^F]**2**); Figure S1: HPLC traces of crude reaction mixture of [^18^F]FE-MPC6827 ([^18^F]**2**); Figure S2: HPLC traces of the purification of [^18^F]FE-MPC6827 ([^18^F]**2**); Figure S3: HPLC traces of purified [^18^F]FE-MPC6827 ([^18^F]**2**) co-injected with the non-radioactive reference compound **2**; Figure S4: Effect of genetic modulation of microtubule stability on [^18^F]FE-MPC6827 ([^18^F]**2**) accumulation; Figure S5: Correlation between changes in [^18^F]FE-MPC6827 uptake and changes in tyrosinated α-tubulin levels observed in HEK293T cells under different experimental conditions; Figure S6: Morphology-based assessment of treatment-associated nuclear changes in HEK293T cells. Additional ^1^H, ^13^C, and ^19^F NMR spectra are also provided.

## Abbreviations

The following abbreviations are used in this manuscript:

[^18^F]F^−^	[^18^F]fluoride
AD	Alzheimer’s disease
ATP	adenosine triphosphate
BBB	blood–brain barrier
Bu_4_NOTs	tetrabutylammonium tosylate
CNS	central nervous system
DIPEA	*N*,*N*-diisopropylethylamine
E_βmax_	maximum positron energy
EOS	end of synthesis
epoD	epothilone D
GFP	green fluorescent protein
HEK293T	human embryonic kidney 293T cells
HPLC	high-performance liquid chromatography
HR-ESI-MS	high-resolution electrospray ionization mass spectrometry
HR-GC-EI/MS	high-resolution gas chromatography electron ionization mass spectrometry
logD_7.4_	distribution coefficient between octanol and buffer (pH = 7.4)
logP	partition coefficient between octanol and water
MARK	microtubule affinity-regulating kinase
MPO	multiparameter optimization
MT	microtubule
n.c.a.	no-carrier-added
NMR	nuclear magnetic resonance
PBS	phosphate-buffered saline
p.c.i.	post-column injection
PD	Parkinson’s disease
PDL	poly-d-lysine
PEG-400	polyethylene glycol
PEI	polyethyleneimine
PET	positron emission tomography
P-gp	P-glycoprotein
PTM	post-translational modification
QMA	quaternary methyl ammonium
r	Pearson’s correlation coefficient
RCC	radiochemical conversion
RCY	radiochemical yield
ROI	region of interest
SEM	standard error of the mean
SPE	solid-phase extraction
spastin	microtubule-severing AAA-ATPase
t_1/2_	half-life
tau	tubulin-associated unit
TFA	trifluoroacetic acid
TLC	thin-layer chromatography
