# Supplementary material for: Radiosynthesis and In Vitro Evaluation of [18F]FE-MPC6827 as a Candidate PET Tracer for Imaging Microtubule Dynamics
Source: Cells. 2026 Nov 7;15(21):2033. doi: 10.3390/cells15212033 (PMC13762428; doi:10.3390/cells15212033)
Supplement: Supplementary file 1 [file cells-15-02033-s001.zip › cells-4646326-supplementary.pdf]

SUPPORTING INFORMATION

Radiosynthesis and *in vitro* Evaluation of [^{18}F]FE-MPC6827 as a PET Tracer Candidate for Imaging Microtubule Dynamics

TABLE OF CONTENTS

1 CNS-MPO, BBB, and CNS-PET-MPO scores 3
2 NMR spectra 4
2.1 ^{1}H NMR spectrum of compound 4 (desmethyl-MPC-6827) 4
2.2 ^{13}C NMR spectrum of compound 4 (desmethyl-MPC-6827) 4
2.3 ^{1}H NMR spectrum of compound 2 (FE-MPC6827) 5
2.4 ^{13}C NMR spectrum of compound 2 (FE-MPC6827) 5
2.5 ^{19}F NMR spectrum of compound 2 (FE-MPC6827) 6
2.6 ^{1}H NMR spectrum of compound 5 6
2.7 ^{13}C NMR spectrum of compound 5 7
3 Radiosynthesis and formulation of [^{18}F]FE-MPC6827 8
3.1 Chromatograms for determination of RCC 8
3.2 Semi-preparative purification of [^{18}F]FE-MPC6827 ([^{18}F]2) 9
3.3 Chromatograms of purified [^{18}F]FE-MPC6827 ([^{18}F]2) 9
4 Uncorrected analysis of fluorescence intensities in transfected HEK293T cells 10
5 Correlation between changes in [^{18}F]FE-MPC6827 uptake and tyrosinated α-tubulin levels observed under different experimental conditions 10
6 Morphology-based assessment of treatment-associated nuclear changes in HEK293T cells 11

1 CNS-MPO, BBB, and CNS-PET-MPO scores

Table S1. CNS-MPO, BBB, and CNS-PET-MPO scores for FE-MPC6827 (**2**)

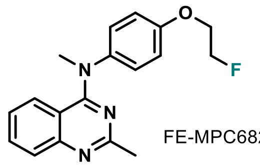

FE-MPC6827 (**2**)

	Value	CNS-MPO	BBB-Score	CNS-PET-MPO
**MW**	311.14	0.79	-	0.86
**TPSA**	38.3	0.91	0.73	0.48
**HBD**	0	1.00	-	1.00
**HBA**	4	-	-	-
**p*K*$_a$**	5.46	1.00	0.45	1.00
**clog*P***	3.42	0.79	-	0.84
**clog*D*$_{7.4}$**	3.33	0.34	-	0.00
**AromR**	3	-	-	-
**HeavyA**	23	-	0.97	-
**MWHBN**	0.23	-	0.99	-
	Total Score:	**5.0**	**4.84**	**3.8**

Parameters were calculated using the ADMETlab platform. MW = molecular weight, TPSA = topological polar surface area, HBD = number of hydrogen bond donors, HBA = number of hydrogen bond acceptors, p*K*$_a$ = ionization constant of the most basic center, clog*P* = calculated partition coefficient, clog*D*$_{7.4}$ = calculated distribution coefficient at pH 7.4, AromR = number of aromatic rings, HeavyA = number of heavy atoms, MWHBN = (MW^(-0.5) × (HBD+HBA)).

2 NMR spectra

2.1 ^{1}H NMR spectrum of compound 4 (desmethyl-MPC-6827)

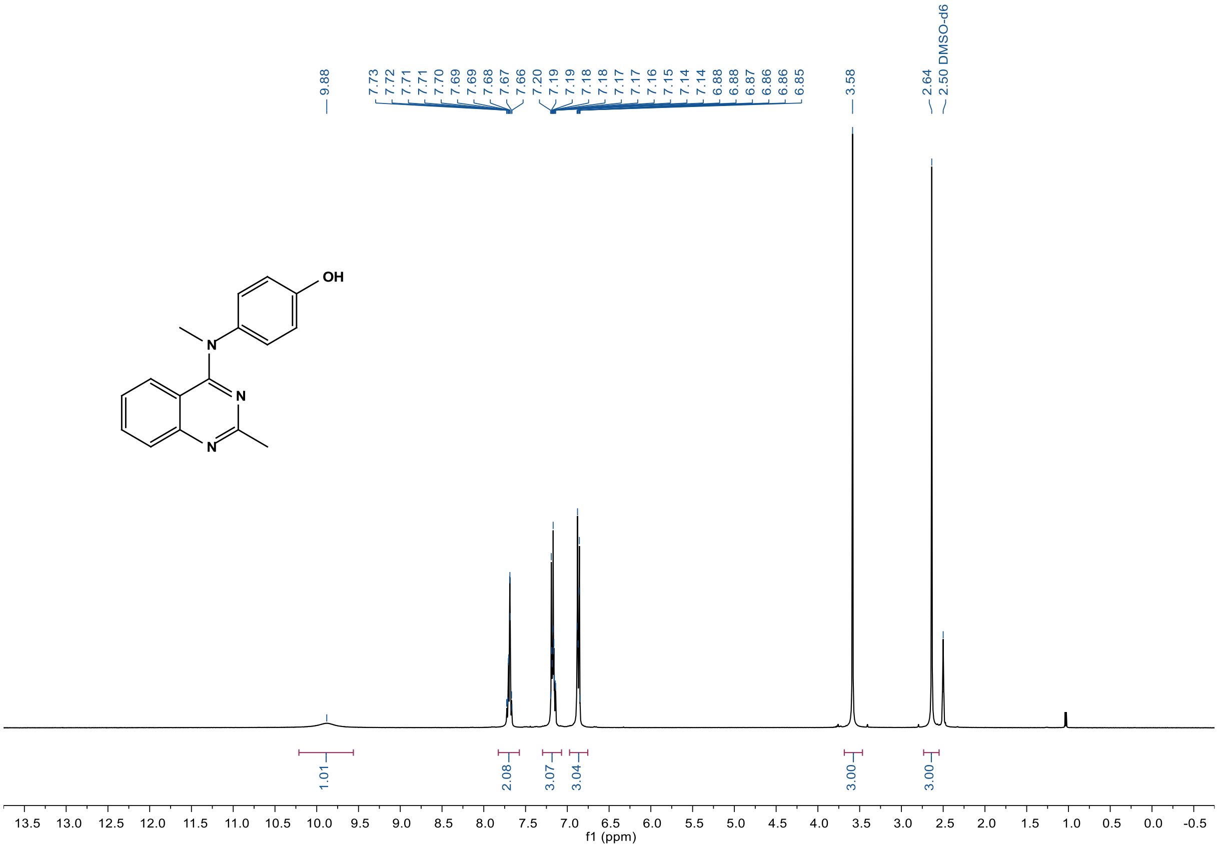

2.2 ^{13}C NMR spectrum of compound 4 (desmethyl-MPC-6827)

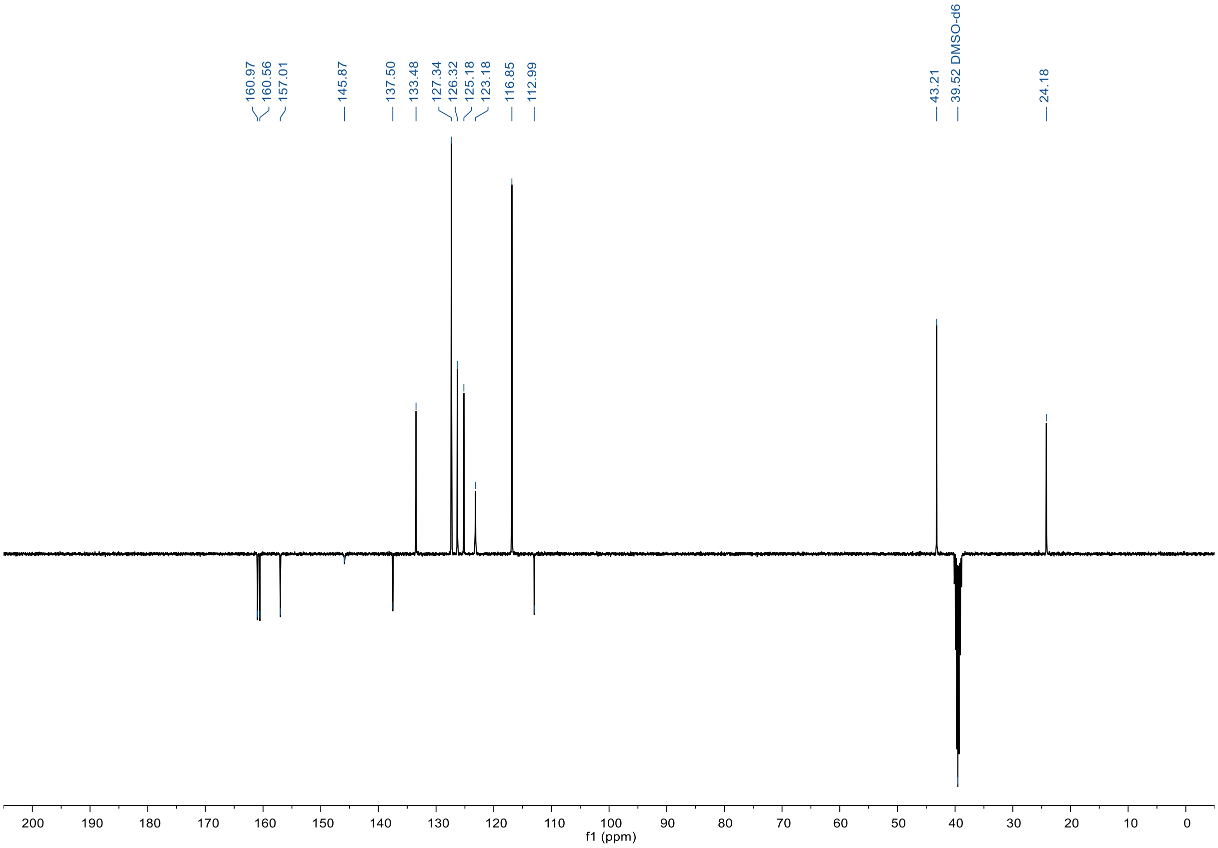

2.3 ^{1}H NMR spectrum of compound 2 (FE-MPC6827)

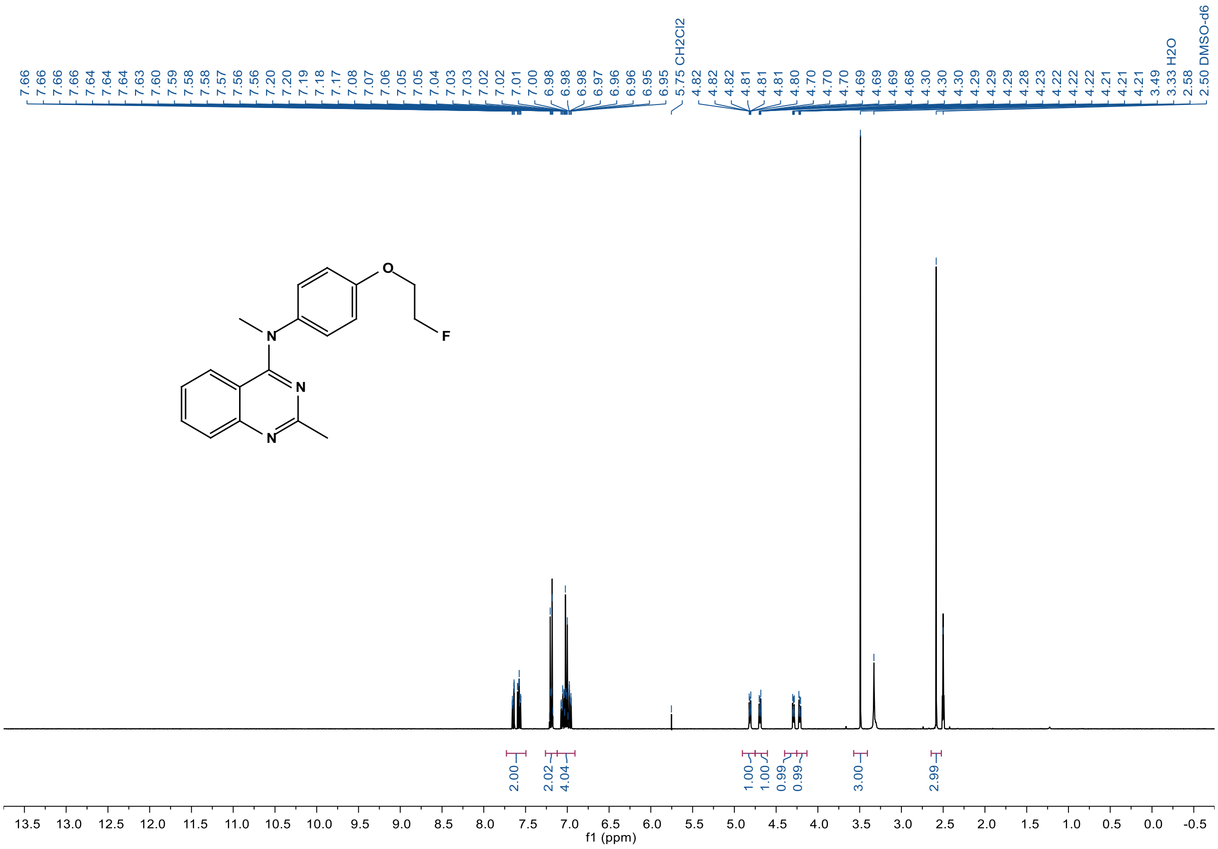

2.4 ^{13}C NMR spectrum of compound 2 (FE-MPC6827)

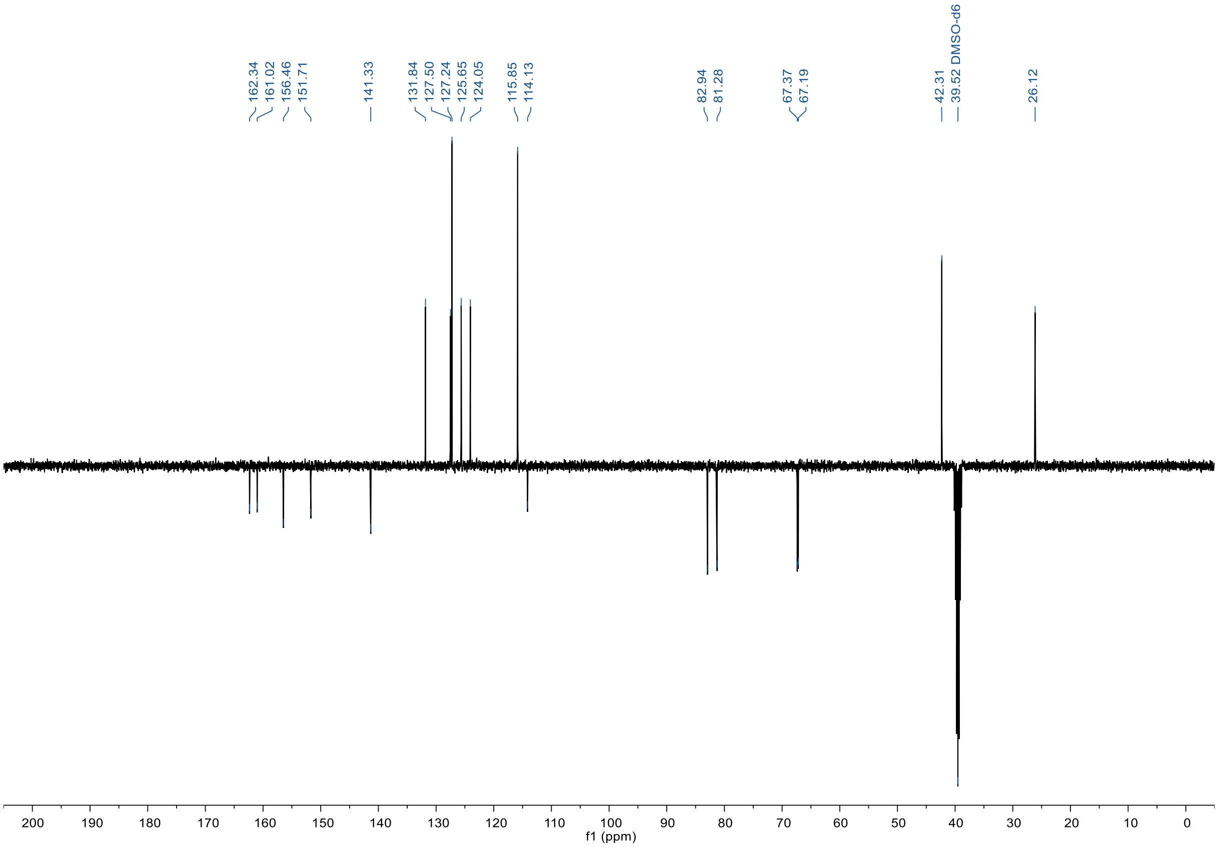

2.5 ^{19}F NMR spectrum of compound 2 (FE-MPC6827)

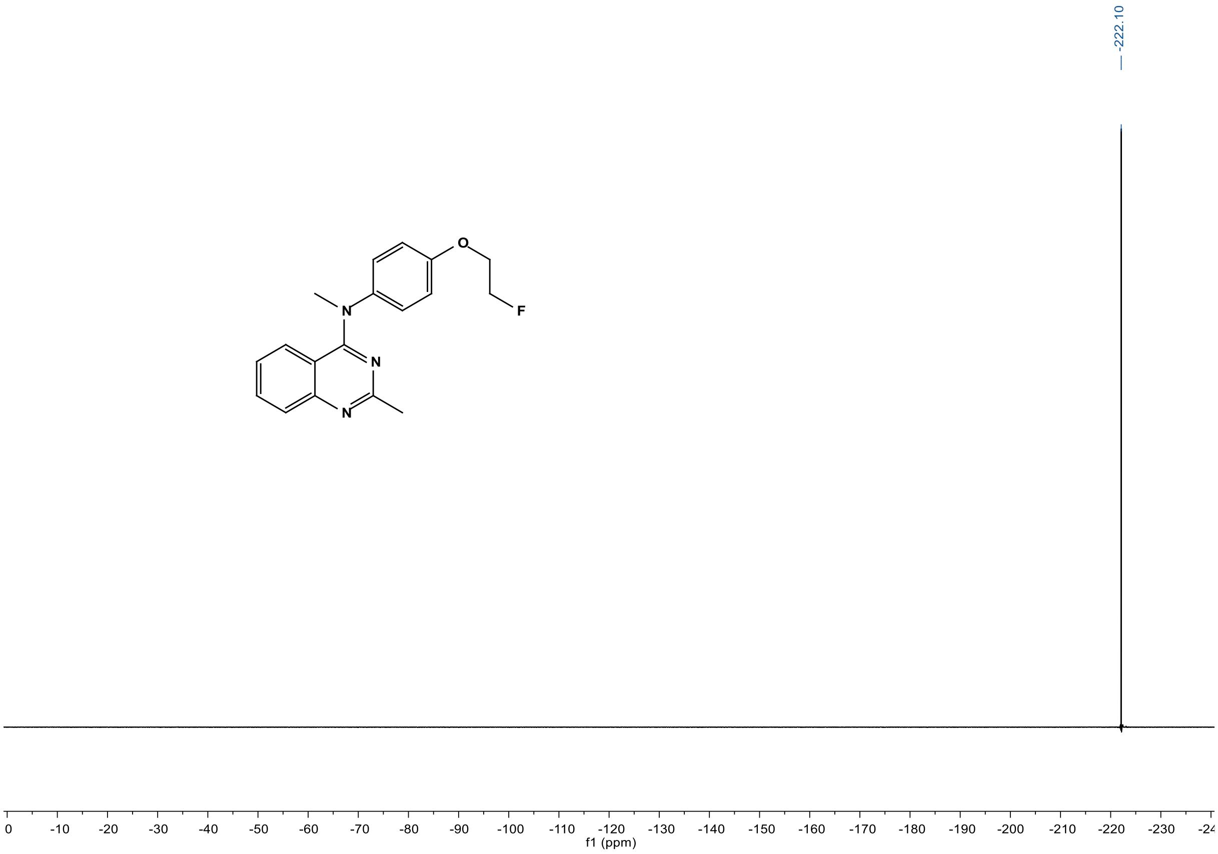

2.6 ^{1}H NMR spectrum of compound 5

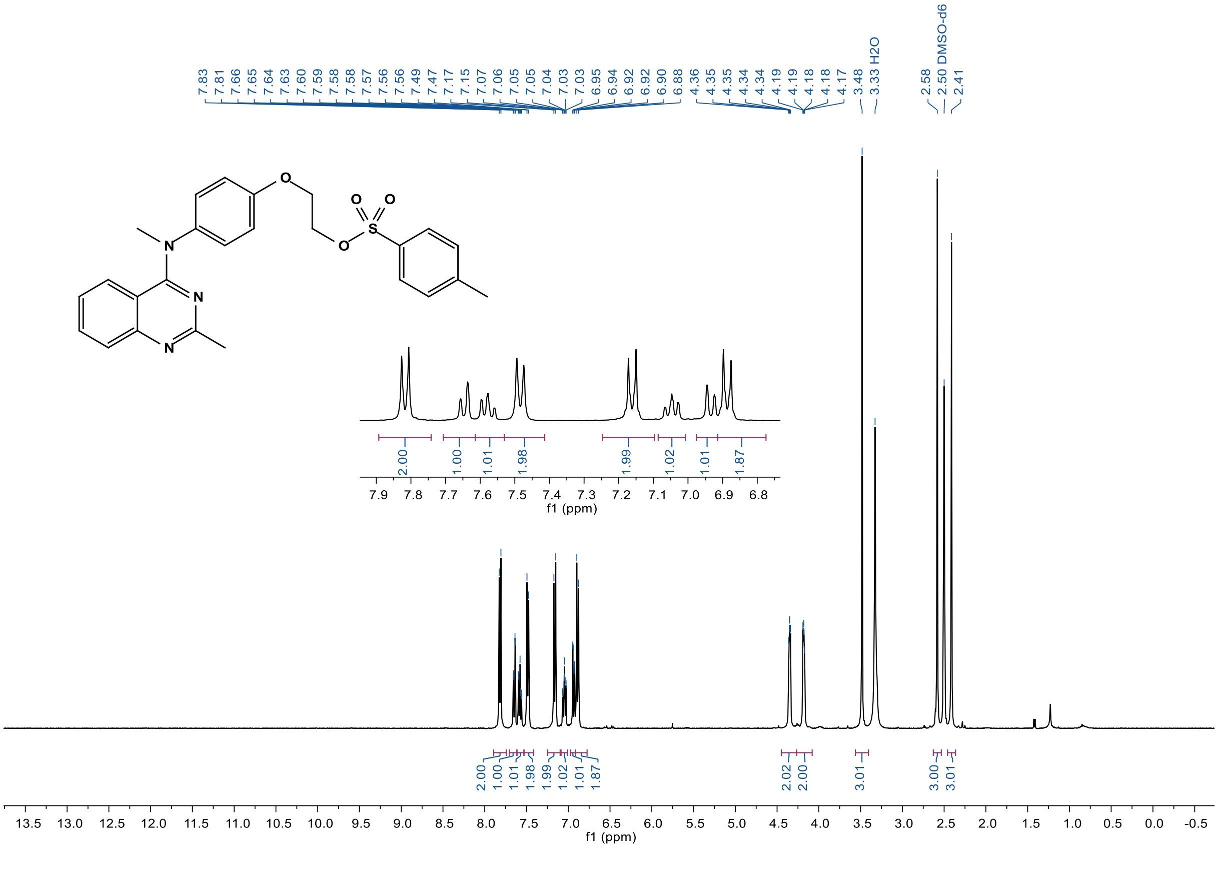

2.7 ^{13}C NMR spectrum of compound 5

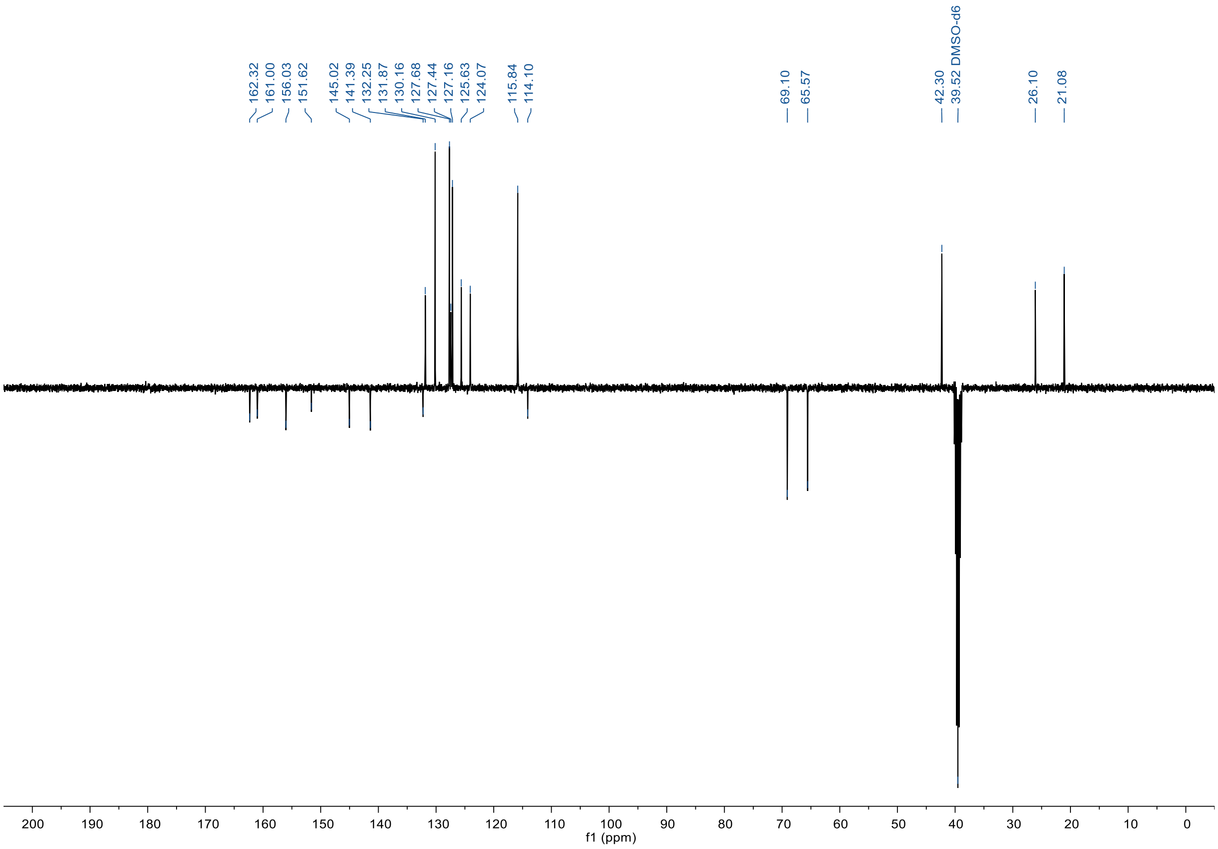

3 Radiosynthesis and formulation of [^{18}F]FE-MPC6827

Scheme S1. Radiosynthesis of [^{18}F]FE-MPC6827 ([^{18}F]**2**)

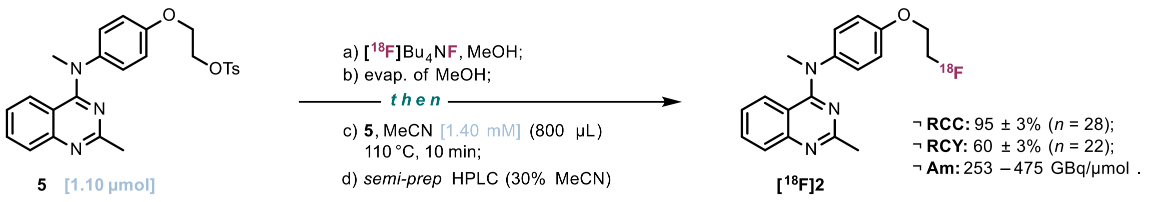

Reagents and conditions: (a) elution of [^{18}F]F$^-$ with Bu_4NOTs (2.42 µmol) in MeOH (1 mL); (b) evaporation of MeOH; (c) addition of precursor **5** (1.10 µmol) in MeCN (800 µL), argon, 110 °C, 10 min; (d) *column*: XBridge BEH Shield RP18, 5 µm (130 Å), 250 ×10 mm (Waters GmbH, Vienna, Austria); *eluent*: 30% MeCN (0.1% TFA); *flow-rate*: 4.7 mL/min. Abbreviations: Bu_4NOTs = tetrabutylammonium tosylate, TFA = trifluoroacetic acid.

3.1 Chromatograms for determination of RCC

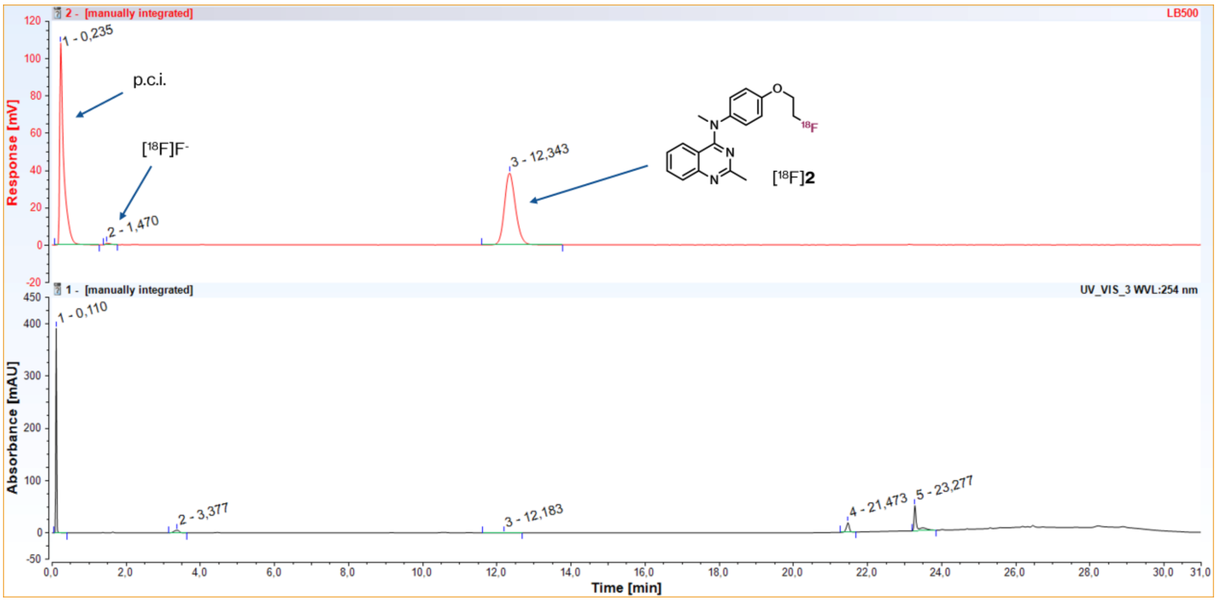

Figure S1. HPLC traces of crude reaction mixture of [^{18}F]FE-MPC6827 ([^{18}F]**2**). Top: radioactivity trace; bottom: UV trace (λ=254 nm). Conditions: *column*: Synergi™ Hydro-RP, 4 µm (80 Å), 250 × 4.6 mm (Phenomenex, Aschaffenburg, Germany); *eluent*: 30% MeCN (0.1% TFA); *flow-rate*: 1 mL/min. Abbreviations: TFA = trifluoroacetic acid, p.c.i. = post-column injection.

3.2 Semi-preparative purification of [^{18}F]FE-MPC6827 ([^{18}F]2)

The crude reaction mixture of [^{18}F]**2** was purified on a semi-preparative HPLC column.

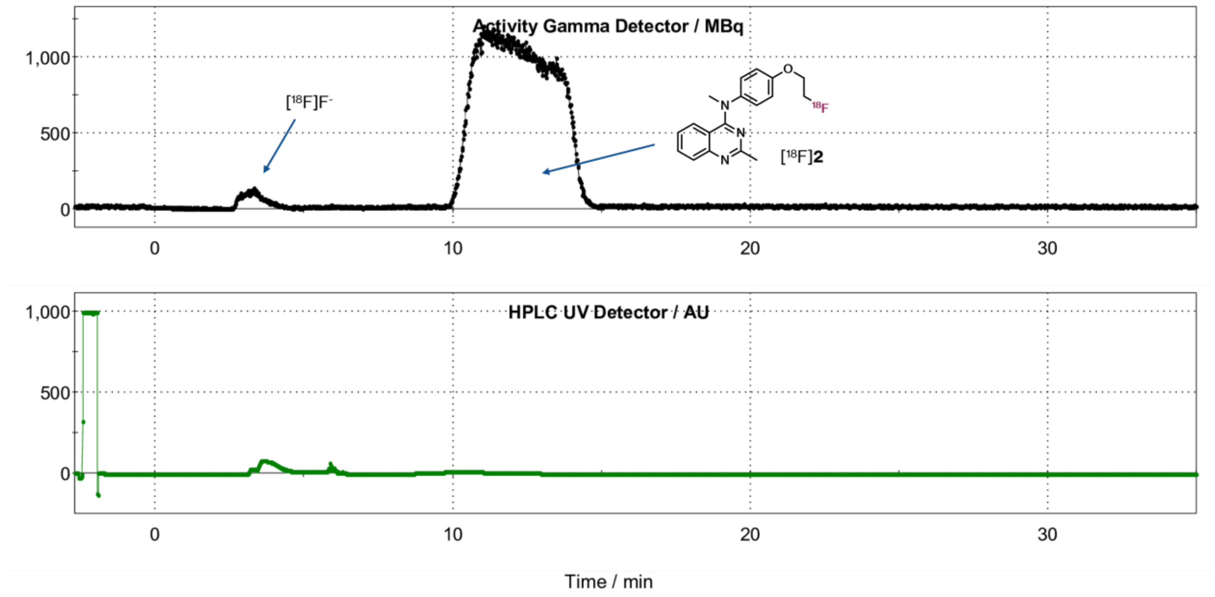

Figure S2. HPLC traces of the purification of [^{18}F]FE-MPC6827 ([^{18}F]**2**). Top: radioactivity trace; bottom: UV trace (λ=254 nm). Conditions: *column*: XBridge BEH Shield RP18, 5 µm (130 Å), 250 × 10 mm (Waters GmbH, Vienna, Austria); *eluent*: 30% MeCN (+ 0.1% TFA); *flow-rate*: 4.7 mL/min. Abbreviation: TFA = trifluoroacetic acid.

3.3 Chromatograms of purified [^{18}F]FE-MPC6827 ([^{18}F]2)

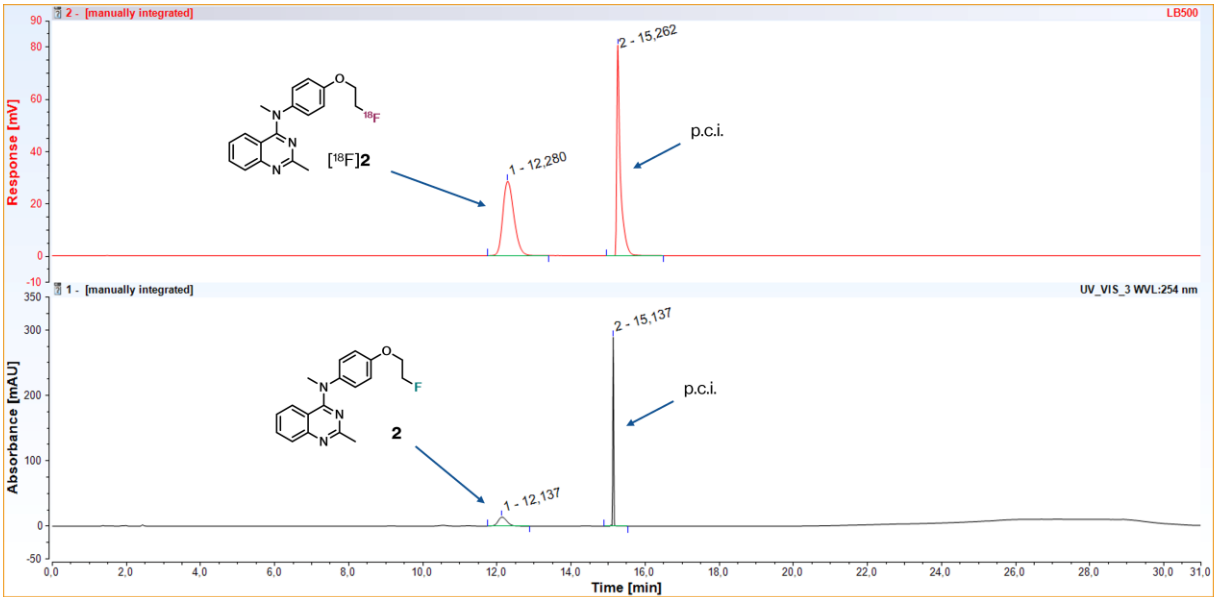

Figure S3. HPLC traces of purified [^{18}F]FE-MPC6827 ([^{18}F]**2**) co-injected with the non-radioactive reference compound **2**. Top: radioactivity trace; bottom: UV trace (λ=254 nm). Conditions: *column*: Synergi™ Hydro-RP, 4 µm (80 Å), 250 × 4.6 mm (Phenomenex, Aschaffenburg, Germany); *eluent*: 30% MeCN (0.1% TFA); *flow-rate*: 1 mL/min. Abbreviations: TFA = trifluoroacetic acid, p.c.i. = post-column injection.

4 Uncorrected analysis of fluorescence intensities in transfected HEK293T cells

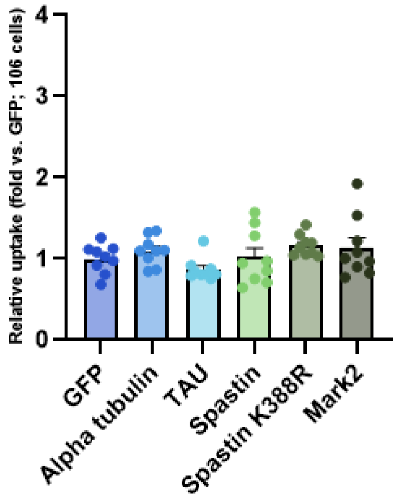

Figure S4. Effect of genetic modulation of microtubule stability on [^{18}F]FE-MPC6827 ([^{18}F]**2**) accumulation. HEK293T cells were transfected with GFP, α-tubulin, TAU, wild-type spastin, ATPase-deficient spastin (K388R), or MARK2, and tracer uptake was quantified after 1 h incubation. Overexpression of α-tubulin, TAU, wild-type SPASTIN, SPASTIN (K388R), or MARK2 did not significantly alter tracer accumulation relative to GFP controls, although a minor downward trend was observed for TAU. Data represent mean ± SEM (3 technical replicates; 3 independent biological experiments). Values were normalized to the mean cell uptake, GFP control, and seeded cell number. No correction for transfection efficiency was applied. Statistical significance was determined using one-way ANOVA with comparison of each construct to GFP controls.

5 Correlation between changes in [^{18}F]FE-MPC6827 uptake and tyrosinated α-tubulin levels observed under different experimental conditions

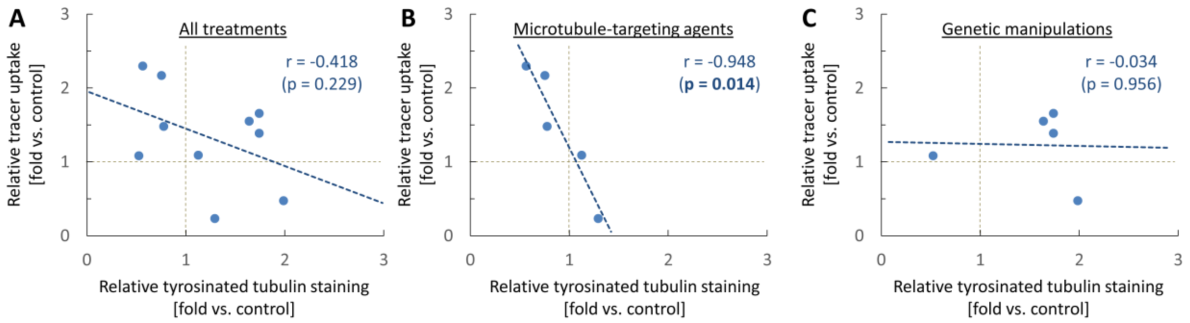

Figure S5. Correlation between changes in [^{18}F]FE-MPC6827 uptake and changes in tyrosinated α-tubulin levels observed in HEK293T cells under different experimental conditions. (**A**) Scatter plot showing the inverse correlation between normalized tracer uptake and tyrosinated α-tubulin fluorescence intensity across all pharmacological and genetic manipulations (excluding homologous blocking with non-radioactive FE-MPC6827). (**B**) Subgroup analysis showing the correlation for the pharmacological treatments only. (**C**) Subgroup analysis showing the correlation for the genetic manipulations only. Each point represents the mean of three biological replicates. Pearson correlation coefficients (r) and *p*-values are indicated in each panel.

6 Morphology-based assessment of treatment-associated nuclear changes in HEK293T cells

To further investigate potential treatment-associated cell death, nuclear morphology was analyzed in fluorescence microscopy images of pharmacologically treated HEK293T cells. Following treatment and fixation, DAPI-stained nuclei were assessed for morphological alterations indicative of cell death. The proportion of affected cells was calculated relative to the total number of nuclei evaluated under each condition. Three replicates were analyzed per treatment condition (n = 3). The corresponding results are presented in Figure S6.

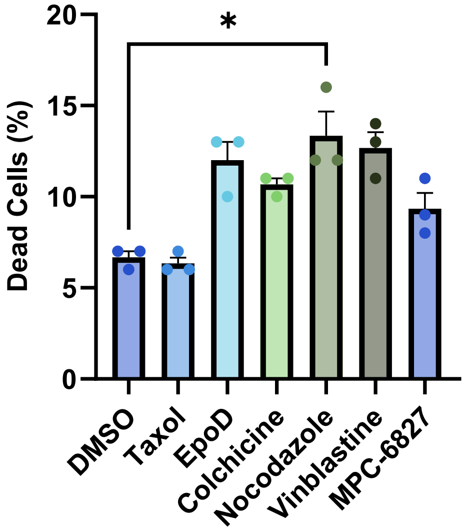

Figure S6. Morphology-based assessment of treatment-associated nuclear changes in HEK293T cells. Cells were treated with DMSO (vehicle control), paclitaxel (Taxol), epothilone D (EpoD), colchicine, nocodazole, vinblastine, or MPC-6827. Nuclear morphology was assessed by fluorescence microscopy, and the percentage of cells exhibiting morphological features indicative of cell death was quantified. Bars represent mean ± SEM, and individual data points represent replicates (n = 3). Statistical analysis was performed using one-way ANOVA, with statistical significance indicated relative to the DMSO control (*$p < 0.05$).
